# Mean-Centering Alters Pathway-Based Transcriptomic Comparisons of Feline and Mouse Kidney Disease

**DOI:** 10.3390/ijms27209115

**Published:** 2026-10-14

**Authors:** Masaki Watanabe, Takeru Sasaki, Ryuya Nakagawa, Nobuya Sasaki

**Affiliations:** Laboratory of Laboratory Animal Science and Medicine, School of Veterinary Medicine, Kitasato University, Towada 034-8628, Aomori, Japan; watanabe.masaki@kitasato-u.ac.jp (M.W.);

**Keywords:** mean centering, pathway aggregation, cross-species comparison, chronic kidney disease, transcriptomics, animal models, cosine similarity, measurement reliability

## Abstract

Transcriptomic similarity is used to characterize disease models, but a comparison can change when the same responses are summarized differently. We examined mean-centering before pathway averaging in 16 disease states from one feline chronic kidney disease cohort and two mouse kidney injury models, using 2016 genes detected in both species’ proteomes and 422 overlapping pathways. Among 87 state pairs without shared control samples, uncentered pathway means separated same-species from cross-species pairs with an area under the curve of 0.868; removing each state’s mean across genes first reduced this to 0.511. The uncentered value was higher in 98.6% of animal-level resamples, and the difference held across nine offset and filter settings, all four gene spaces and the 83-pair subset without shared animals. Under within-state ranking or quantile normalization, which equalize each state’s overall level by construction, the contrast fell to 0.010 and 0.001. Reference-axis projections can rank states differently from cosine because they retain amplitude, and benchmark gaps were poorly calibrated. These results identify a consequential preprocessing choice rather than a preferred species or a validated measure of disease fidelity. Because all feline states came from one cohort and all mouse states from two datasets, species was confounded with dataset throughout, and the separation reported here may reflect dataset-specific technical variation rather than species biology.

## 1. Introduction

Animal models must be selected before their ability to predict a therapeutic response in patients is known. In kidney disease, that decision has prompted guidance on the design and interpretation of preclinical studies [1,2,3]. Transcriptomic comparisons can contribute molecular evidence, but their value depends on a prior question: which feature of the disease response does a similarity score retain? A high score can describe agreement in the direction of expression changes, their amplitude, or a selected set of pathways. Those features need not favor the same model.

Animal models of kidney disease have been developed separately in each species and for different injuries. In rodents, surgical, toxic, immune and genetic models each reproduce part of the human disease and are chosen for the mechanism they isolate rather than for overall resemblance [4]. Reviews of the field have noted that no rodent model reproduces the slow progression of human chronic kidney disease, and that the strain, sex and injury used change what the model shows [5,6]. Naturally occurring disease in companion animals has been proposed as a complement for that reason, since it arises without intervention and progresses over years [7]. Models have most often been compared by the phenotypes they produce—histology, function, time to failure—and molecular comparison between species has been added later, which is where the present question arises.

Cross-species comparisons have used rank correlation, overlap of differentially expressed genes, pathway concordance and trajectory alignment. Analyses of the same inflammatory-disease data have reached opposing conclusions about model similarity [8,9], illustrating how analytical choices can affect interpretation. We focus here on a specific choice within pathway averaging: whether each state’s average response across genes is removed before pathway means are compared.

Pathway analysis offers a biologically organized description of a complex expression response. Congruence analysis, for example, measures agreement between model and human responses within pathways [10]. In a different construction, the mean response in each pathway becomes one coordinate of a pathway-level vector. Subtracting a state’s mean across the selected genes changes that vector from a summary of the original response to a summary of deviations from its average. This distinction can matter even when the input genes, samples and pathway definitions remain unchanged.

Pairwise and reference-anchored comparisons also ask different questions. Time-course methods align expression trajectories [11], while gene-level probabilistic alignment identifies where one trajectory follows or departs from another [12]. Alignment with one fixed disease profile is simply a selected row of the pairwise similarity matrix. States may form an orderly time course without progressively approaching that reference.

Projection scores retain both the magnitude and direction of a response. The signature projection score was introduced for drug signatures [13,14], while later cross-species methods used latent-space projection or learned mappings between species [15,16]. Retaining amplitude can be useful when a large and a small response in the same direction have different biological meanings. The decomposition of a projection into amplitude and directional alignment is elementary. Our purpose is to illustrate its consequences for ranking disease states, not to introduce a new similarity measure.

This issue is specific to unnormalized projections. Cosine similarity and rank correlation are invariant to positive rescaling of either profile, although they differ in other properties and need not give the same ordering. Neither has been established here as a superior predictor of biological fidelity.

Feline chronic kidney disease provides a naturally occurring disease reference. It is common in older cats [17], and its tubulointerstitial inflammation and fibrosis have motivated its use as a model of renal interstitial fibrosis [7]. We compared four feline cortical and medullary CKD states, three Pod-TRECK podocyte injury time points and nine ischemia–reperfusion injury time points. The main reference was feline cortical CKD3/4. All comparisons used a common gene space and the difference in mean log2 expression between each disease group and its assigned controls.

Our main question was how centering before pathway averaging changed the comparison of same-species and cross-species state pairs. We then examined sensitivity to gene selection and preprocessing, separated amplitude from direction in reference-axis projections, and assessed shared controls and reliability-derived benchmarks. These supporting analyses clarify what the scores can establish; they are not additional tests of a species effect.

All feline states came from one cohort and the mouse states from two model datasets. Species could not be separated from dataset, platform or sampling differences. The study is therefore a methodological case study in kidney disease, with exploratory time-course and human comparisons used to examine the scope of its conclusions.

## 2. Results

### 2.1. Mean-Centering Changes Pathway-Level Comparisons

We defined a disease state as one disease group contrasted against one control group within a single dataset and a single measurement layer, and assembled 16 such states on a common human gene space: feline cortical and medullary CKD1/2 and CKD3/4, Pod-TRECK at days 5, 14 and 21, and nine ischemia–reperfusion injury (IRI) time points from 2 h to 12 months. Each state is a vector of differences in mean log2 expression against its own control group (Section 4.4). Unless stated otherwise, all analyses below are restricted to Group A, the genes detected in both the feline and the mouse proteomes (2240 genes; 2016 with finite values in all 16 states).

**Figure 1:**
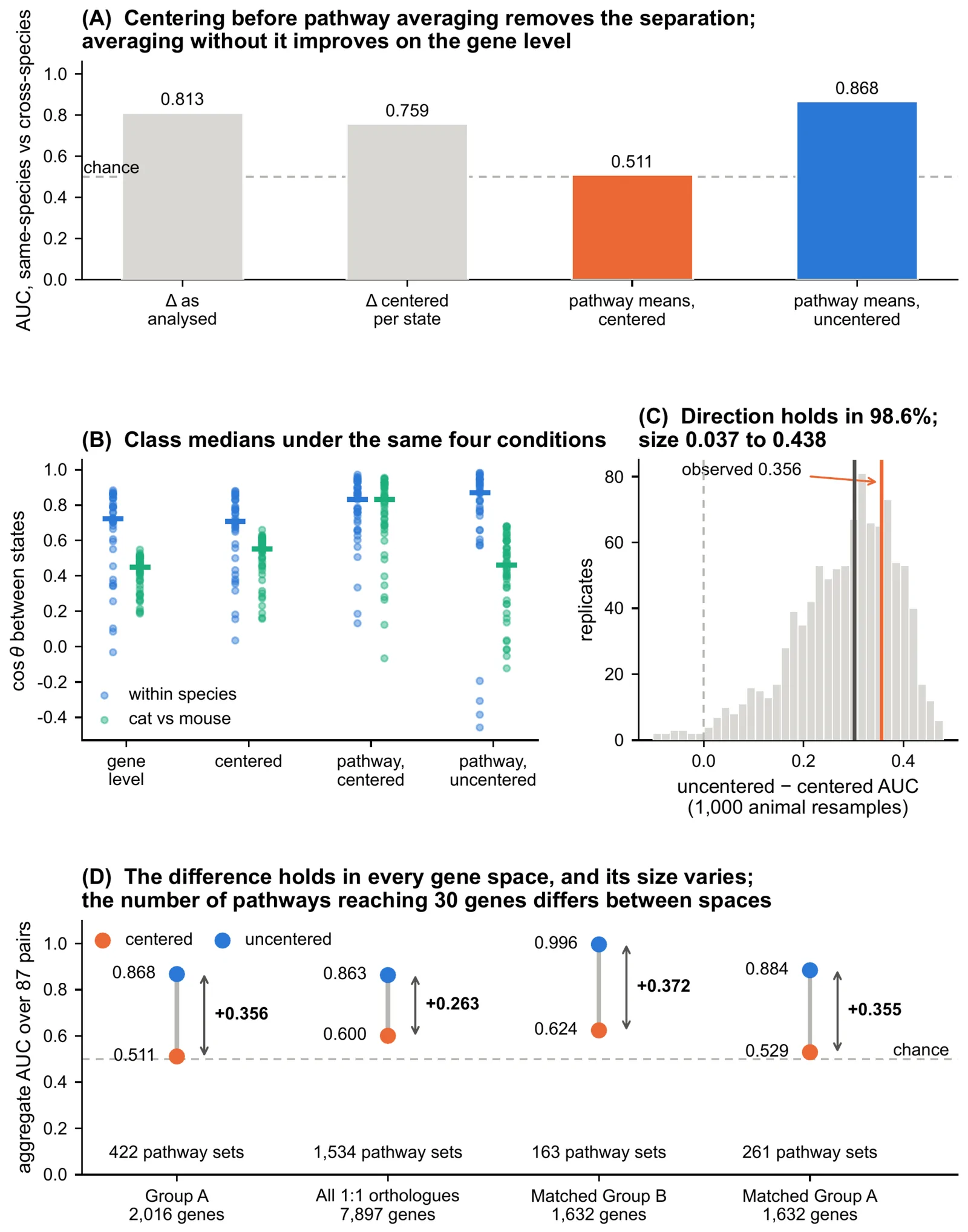
Effect of mean-centering and pathway averaging on pairwise similarity. (**A**) Descriptive AUC for separating 39 same-species from 48 cross-species pairs without shared control samples. (**B**) Cosine of every pair in each class under the same four conditions, with class medians marked. (**C**) Distribution of the uncentered-minus-centered aggregate AUC over 1000 animal-level resamples, with the observed difference and the resampled median marked. (**D**) Centered and uncentered aggregate AUC in each of the four gene spaces of Table 1, over the same 87 pairs, with the difference between them. The number of pathways reaching the 30-gene threshold differs between spaces and is given beneath each pair of points; the matched Group B value rests on 163 sets against 422 in Group A. The four spaces are not independent, since three are drawn from the same transcriptome as the first. Centering subtracts each state’s mean across the common gene universe before pathway averaging. Positive scalar standardization does not alter cosine. These dependent pair-class AUCs are not independent test-set accuracies; animal-resampling summaries are reported in Section 2.1.

The primary pathway analysis used 422 sets containing at least 30 Group A genes: 229 Reactome [18], 118 Gene Ontology Biological Process [19], 48 KEGG [20] and 27 Hallmark [21] sets. We compared cosine similarity between gene-level response vectors with cosine similarity between vectors of pathway means. For each representation, we assessed whether subtracting each state’s mean across the common gene universe changed the separation of pair classes (Figure 1).

**Table 1:** Reported results in four gene spaces. The matched columns contain different genes linked by the same 1632-pair matching. Mantel rows use cosine dissimilarity. The 75-pair AUCs use cross-dataset pairs; pathway-specific and centered aggregate AUCs use 87 pairs without shared control samples. Benchmark exceedances refer to point estimates, and state-bootstrap ranges are descriptive. Uncentered aggregate AUCs are given for all four spaces, so the table shows whether the centering contrast holds outside Group A. The number of qualifying pathway sets differs between spaces because the 30-gene criterion is applied within each space.

Quantity	Group A (2016)	All 1:1 Orthologues (7897)	Matched Group B (1632)	Matched Group A (1632)
Feline cortical CKD1/2: α	0.544	0.604	0.546	0.566
Feline cortical CKD1/2: cos θ	0.907	0.898	0.876	0.901
Feline cortical CKD1/2: ν	0.600	0.673	0.623	0.629
Feline medullary CKD1/2: α	1.020	0.796	0.987	0.947
Feline medullary CKD1/2: cos θ	0.790	0.730	0.734	0.776
Feline medullary CKD1/2: ν	1.291	1.091	1.345	1.221
α and cos θ ordered oppositely (the two states above)	yes	yes	yes	yes
ν needed for α > 1, i.e., 1/cos θ (medullary)	1.266	1.371	1.362	1.289
margin, ν − 1/cos θ (medullary)	+0.025	−0.280	−0.017	−0.069
α above the reference’s own 1.000	yes	no	no	no
Mantel time statistic using cosine dissimilarity	+0.599	+0.637	+0.646	+0.613
Mantel, elapsed time, direction (permutation *p*)	0.0040	0.0011	0.0009	0.0031
cos θ against log time, per state	+0.074	+0.627	+0.147	+0.210
State-bootstrap range lower percentile	−0.72	0.00	−0.61	−0.55
State-bootstrap range upper percentile	+0.72	+0.96	+0.77	+0.78
Cat–mouse cos θ, maximum	0.548	0.572	0.438	0.534
Lowest Spearman–Brown benchmark, cat–mouse	0.765	0.780	0.765	0.769
Cat–mouse pairs above their benchmark of 48	0	0	0	0
AUC over the 75 cross-dataset pairs (no permutation test, Section 2.5)	0.7909	0.7616	0.7708	0.8102
Pathway-level AUC, median	0.760	0.782	0.780	0.781
AUC after aggregation, centered (Section 2.1)	0.511	0.600	0.624	0.529
AUC after aggregation, uncentered (Section 2.1)	0.868	0.863	0.996	0.884
Uncentered minus centered aggregate AUC	0.356	0.263	0.372	0.355
Pathway sets with at least 30 genes	422	1534	163	261

The principal comparison comprised 87 state pairs without shared control samples: 39 same-species and 48 cross-species pairs. Throughout, these labels describe the sampling design rather than a biological attribution. Every cross-species pair joins the feline cohort to a mouse dataset, so the two classes differ in dataset, platform and processing as well as in species, and a separation between them is equally a separation between the sampled datasets. Four feline cortical–medullary pairs nevertheless shared control animals across tissues; their exclusion was examined separately. AUC describes how well similarity separates these pair classes, which reuse states and animals. It is not a measure of independently validated classification performance (Table S1).

Uncentered pathway means gave an AUC of 0.868, compared with 0.511 when each state’s mean across genes was removed before pathway averaging. The whole-gene AUC was 0.813 and fell to 0.759 after centering alone. Centering therefore changed the AUC by 0.054 at gene level and by 0.356 after pathway averaging, almost seven times as much; what is consequential is the combination of the two steps, not either one alone (Figure 1A).

Centering and scaling have different effects: subtracting a state’s mean response across genes changes its direction, whereas dividing by a positive standard deviation leaves cosine unchanged before and after pathway averaging, so the cosine between centered gene vectors is the Pearson correlation between the original vectors (Section 4.13). The relevant choice is therefore whether to remove each state’s mean before taking pathway averages.

The class medians show how the representations differed (Figure 1B). At gene level, same-species and cross-species median cosines were 0.724 and 0.448; after centering, they were 0.709 and 0.551. Uncentered pathway means gave medians of 0.870 and 0.462, whereas centered pathway means gave nearly identical medians of 0.831 and 0.832. The near-chance AUC of the latter representation describes this panel and does not establish that all biological information was lost. The IRI 12-month state is the only one contrasted against an age-matched control group and contributes 15 of the 87 pairs; excluding it leaves 72 pairs with a whole-gene AUC of 0.798, an uncentered aggregate AUC of 0.858 and a centered one of 0.546, so the centering difference falls from 0.356 to 0.312. The contrast is not carried by that state, but part of its size is. The control assignments of Section 4.5 do not remove all age differences between states.

In 1000 animal-level resamples preserving shared controls and cortical–medullary pairing, the uncentered pathway AUC exceeded the centered AUC in 98.6% of replicates. The median difference was 0.302, with a central 95% range of 0.037–0.438. The corresponding ranges were 0.724–0.899 for the uncentered AUC and 0.432–0.737 for the centered AUC. These are descriptive resampling ranges, not calibrated confidence intervals. After excluding the four additional pairs sharing control animals across tissues, the remaining 83 pairs had a whole-gene AUC of 0.792, a median pathway-specific AUC of 0.744, a centered aggregate AUC of 0.491 and an uncentered aggregate AUC of 0.852. The centering difference in this subset, 0.361, is close to the 0.356 obtained in the full set of 87 pairs, so the contrast does not depend on the four pairs that share animals across tissues. Twelve of the 87 pairs are within-dataset as well as same-species: those four feline pairs and eight between IRI 12 months and the earlier IRI time points. Removing both groups leaves the 75 pairs that share neither controls nor a dataset (27 same-species and the same 48 cross-species pairs; Table S1), for which the uncentered aggregate AUC is 0.856 against 0.518 centered, a difference of 0.337. Under the same animal-level resampling the uncentered value exceeds the centered one in 98.7% of replicates, with a median difference of 0.284 and a central 95% range of 0.055 to 0.420.

The decomposition explains why. A pathway mean is the sum of two parts: the state’s mean change across all measured genes, which every pathway of that state shares, and the pathway’s own deviation from it. Uncentered pathways means are dominated by the shared part, because it enters all 422 coordinates with the same sign, and that common level differs between the feline and the mouse states. Averaging within pathways also removes much of the gene-level noise that would otherwise dilute it, which is why the uncentered aggregate separates the classes better than the gene-level comparison it replaces. That common level is measurable, and the two species do not overlap in it (Table S2): the mean Δ ranges from −0.325 to −0.088 in the four feline states and from −0.068 to +0.426 in the twelve mouse states, so every feline state sits below every mouse state, the closest pair being feline cortical CKD1/2 at −0.088 against IRI 2 h at −0.068. A single number per state therefore separates the species here, which is what makes the retained mean so consequential after averaging. That number is also the component of a response most directly exposed to differences in platform, normalization and expression units between datasets (Section 4.4), and the four feline states come from one cohort and one deposited dataset. Their common negative mean is therefore consistent with a global feature of feline CKD and equally with a cohort-level property of how the data were generated; the design cannot distinguish the two. Centering removes the shared part first, leaving only the relative differences between pathways, and those relative patterns are similar in cat and mouse: both classes rise to a median near 0.83 and the separation goes to chance. At the gene level the same subtraction moves little, because a common shift is one direction among 2016 rather than one among 422 pathways, which means that all carry it.

The response vector can be defined in other ways, and the comparison depends on which is used. We repeated the whole 16-state analysis under three alternatives to the difference in mean log2 expression: standardizing each gene across the 16 states, ranking the genes within each state, and quantile-normalizing the states to a common marginal distribution (Table S3). The mean Δ separates the species under the first alternative as well as under the main definition; the feline states run from −0.856 to −0.415 against −0.373 to 0.924 in the mouse states. Under ranking and quantile normalization it does not, but that is a consequence of the definitions rather than an independent finding: both give every state the same marginal distribution, so the mean over genes is identical for all sixteen by construction (0.500 under ranking, 0.0789 under quantile normalization). The centering contrast follows the same order. It is 0.868 against 0.511 under the main definition, 0.612 against 0.470 with genes standardized, and 0.490 against 0.480 and 0.491 against 0.490 under ranking and quantile normalization, so the difference falls from 0.356 to 0.143, 0.010 and 0.001.

That pattern is what the mechanism predicts. If the separation carried by the uncentered pathway means it comes from a state-wide level entering all 422 coordinates with the same sign, a definition that equalizes that level across states should reduce the gap, and it does: under ranking, the uncentered aggregate cosines collapse onto one another, with medians of 0.9914 within and 0.9931 across species and a total spread of 0.041 over all 87 pairs, and the aggregate AUC falls to 0.490, at chance, close to the centered value of 0.480. The remaining differences of 0.010 and 0.001 are measured rather than zero by construction, because subtracting a per-state constant still changes a cosine taken after pathway averaging. The conclusion that centering changes the answer therefore holds for response definitions that retain each state’s overall level, which the difference in mean log2 expression and the per-gene standardization both do. The main analysis keeps the difference in mean log2 expression; the three alternatives are sensitivity analyses, not competing primary definitions.

Pathway activity is also quantified by methods that score each state from the ranks of its genes rather than from their values, and these behave as the mechanism predicts. We recomputed the same 87-pair comparison over the same 422 sets with the Bioconductor implementations of ssGSEA [22], GSVA [23], singscore [24] and PLAGE [25] (Section 4.14, Table S4). Under ssGSEA the uncentered and centered aggregates give 0.477 and 0.477, a difference of 0.000; under singscore 0.482 and 0.482; under GSVA 0.472 and 0.514; and under PLAGE 0.642 and 0.568. For ssGSEA and singscore the two score matrices are identical, because subtracting one constant from every gene of a state leaves the ranking of that state’s genes unchanged and these methods use only that ranking. What changes across methods is therefore not which representation wins but whether either separates the classes at all: the uncentered value falls from 0.868 with pathway means to about 0.48 with the rank-based methods, while the centered value stays near chance throughout. PLAGE, which standardizes genes within each set across states and so removes part but not all of the state-wide level, sits between the two.

The centering contrast is therefore specific to aggregation by arithmetic means of values that retain each state’s overall level. Two operations at different stages of the analysis remove it in the same way: defining the response by ranks rather than by differences in mean log2 expression (Table S3), and scoring pathways by ranks rather than by means (Table S4). Both point to the same component, and neither identifies a representation that is biologically more faithful.

Averaging is part of the mechanism but does not account for all of it. Repeating the aggregation on 300 sets of 422 random gene sets matched to the sizes of the real ones, the centering difference was positive in every draw, with a median of 0.163 (range 0.097–0.217). The observed 0.356 lies above every one of those draws, and the random sets also behave differently in level: their centered aggregate AUC stays near 0.76 (0.705–0.832) rather than falling to chance, while their uncentered value reaches 0.92. Matching sizes does not, however, preserve the overlap between sets, and the 422 real sets reuse genes heavily: a gene belongs to a median of 7 of them and one to 184, while 130 of the 2016 belong to none. A second control, therefore, kept the 422 real sets and permuted the gene labels of the Δ matrix, which preserves set sizes, every pairwise overlap and the reuse count of every gene, and breaks only the association between gene identity and set membership. Across 300 such draws, the difference had a median of 0.189 and a much wider spread (−0.055 to 0.385), reaching or exceeding the observed 0.356 in 3 of them; the centered aggregate AUC fell to 0.55 or below in 3 draws and to 0.501 in 1 draw, below the observed centered value of 0.511. Averaging any 422 coordinates therefore reproduces part of the contrast, and preserving the size and overlap structure of the real collection reproduces more of it, occasionally all of it, so we do not claim that the near-chance centered score is unique to the real pathway sets. The retained mean may reflect disease regulation, cellular composition or assay properties, and stronger separation of the sampled pair classes does not establish that the uncentered score is biologically preferable.

### 2.2. Sensitivity to Gene Selection and Preprocessing

Proteomic detection restricted the primary analysis to Group A. This selection did not use the sign of the transcriptomic disease response, but favored highly expressed genes and was not functionally neutral. We repeated selected analyses in all one-to-one orthologues (7897 genes), expression-matched Group B (1632 genes) and the corresponding matched Group A (1632 genes; Table 1). The matched sets contained different genes linked by one deterministic pair list. The standardized mean expression-rank difference was −0.017 after matching, compared with +1.236 before matching; 384 Group A genes lacked a partner within the caliper.

Centered aggregate AUCs were 0.511, 0.600, 0.624 and 0.529 in Group A, all one-to-one orthologues, matched Group B and matched Group A, respectively, and the corresponding uncentered aggregate AUCs were 0.868, 0.863, 0.996 and 0.884 (Table 1). The uncentered value exceeded the centered one in every space, by 0.356, 0.263, 0.372 and 0.355, so the centering contrast is not specific to the primary gene set. Two qualifications attach to it. First, the four spaces do not contain the same pathways: the number of sets reaching 30 genes is 422, 1534, 163 and 261, so a comparison across spaces confounds the change in genes with the change in pathway set. Restricted to the 117 pathway identifiers that qualify in every space, the differences become 0.358, 0.381, 0.388 and 0.328 (Table S5), so part of the spread between spaces is explained by which pathways qualify in each; a difference remains over the common sets, and a pathway of the same name contains different genes in different spaces, so the gene space still enters through the membership of each set. Second, the matched Group B value rests on only 163 pathways, because far fewer sets reach 30 genes in that space, and its near-complete separation (0.996) should be read with that in mind. The number of coordinates does not by itself produce such a value: drawing 163 of the 422 Group A set at random, 300 times, left the uncentered aggregate AUC at a median of 0.866 (0.844–0.888) against 0.868 for all 422, and no draw reached 0.996, so the matched Group B value reflects that gene space rather than the smaller number of coordinates. Median pathway-specific AUCs exceeded the centered aggregate AUC in every space.

The 422 sets are drawn from four collections that overlap, and more than half are Reactome sets, so we asked whether the contrast depends on that composition (Table S5). Taken one collection at a time, it does not: the uncentered aggregate exceeds the centered one by 0.294 over the 229 Reactome sets, 0.388 over the 118 GO Biological Process sets, 0.330 over the 48 KEGG sets and 0.444 over the 27 Hallmark sets, against 0.356 over all four together. Removing redundant sets does not reduce it either. Keeping one representative of each group of sets whose pairwise Jaccard overlap reaches 0.5 leaves 221 of the 422, and the difference is 0.382; at thresholds of 0.3 and 0.2 it is 0.387 over 159 sets and 0.377 over 119. The contrast is slightly larger among the non-redundant sets than among all of them.

Other results also depended on gene selection. The two feline CKD1/2 states had opposite projection and cosine orderings in all four spaces, but the medullary projection exceeded one only in Group A. Cross-dataset pair-class AUCs ranged from 0.7616 to 0.8102. Time associations and descriptive reliability benchmarks are included in Table 1 and the Supplementary Notes. None of these checks supplies an independent cohort replication.

We also varied the log2 offset (1, 0.5 or 0.1) and the group-mean FPKM threshold (1, 0 or 5; Table S6). Across nine combinations, whole-gene AUC ranged from 0.813 to 0.821, uncentered pathway AUC from 0.863 to 0.877 and centered pathway AUC from 0.510 to 0.545. The uncentered-minus-centered difference ranged from 0.326 to 0.357, calculated before rounding. The opposite α and cosine ordering of the feline CKD1/2 states, and the medullary point estimate above α = 1, were retained in all nine settings. These nine settings vary the offset and the expression filter within Group A; they are not additional gene spaces. These checks support sensitivity conclusions within the tested settings, not invariance to other normalization methods.

Orthologue and gene-selection checks are summarized in Supplementary Note S3, Table S7 and Figure S1. Sequence-identity restriction did not remove the pair-class difference, while expression matching attenuated the Group A–Group B correlation difference. Neither procedure isolates a technical or biological cause.

### 2.3. Pathway-Specific Alignment

Individual pathways gave a wider range of alignments than the whole-gene comparison. The median pathway-specific AUC was 0.760 (interquartile range 0.707–0.808); 23% of pathways reached the whole-gene value of 0.813. The random-set background also widened as gene count decreased: its central 95% cosine range spanned 0.652 at 30 genes, 0.540 at 50 and 0.419 at 100 (Figure S2A). These ranges describe variation among gene sets, not sampling uncertainty for a particular biological pathway.

Three selected focal-adhesion comparisons illustrate local alignment: IRI 7 d, 72 h and 28 d had cosines of 0.935, 0.912 and 0.902 over 53 genes. They were identified by screening 2136 mouse-state-by-pathway cells from sets with at least 50 genes. Their animal-resampling summaries are given in the Supplementary Notes. Neither that resampling nor the size-matched random-set comparison accounts for post hoc selection, so the examples do not establish a focal-adhesion-specific model advantage.

Rankings of the twelve mouse states varied across pathways (Figure S3A; Kendall’s W = 0.320). The median pairwise rank correlation was 0.336. IRI 28 d led 22% of sets, IRI 12 mo led 21% and IRI 2 h led 11%, but these shares repeatedly count overlapping processes: 47 of the 48 sets led by IRI 2 h were Reactome sets, many related to the proteasome. They cannot be used to identify an overall best model. Collection-specific and reduced-overlap analyses of the kind applied to the centering contrast in Section 2.2 would be needed to separate pathway membership from repeated weighting of shared genes in these leading-share counts, and we did not carry them out for this statistic.

### 2.4. Reference-Axis Projections Retain Amplitude as Well as Direction

Taking the feline cortical CKD3/4 response as reference vector a and another state as v, we defined the normalized projection α, cosine alignment cos θ, relative amplitude ν and normalized residual R⊥ as follows:

$$\begin{array}{rrrr} \alpha & =\frac{a\cdot v}{a\cdot a}, & \cos\theta & =\frac{a\cdot v}{\left\|a\right\|\left\|v\right\|},\\ \nu & =\frac{\left\|v\right\|}{\left\|a\right\|}, & R_{⊥} & =\frac{\left\|v-\mathrm{\alpha a}\right\|}{\left\|v\right\|} \end{array}$$

These quantities satisfy the identities

(1) $$\alpha =\nu \cos\theta ,\;\;R_{⊥}=\sqrt{1-\cos^{2}\theta }=\sin\theta .$$

Thus, α combines directional alignment with relative amplitude. The residual depends only on the magnitude of the cosine and discards its sign. It does not provide an independent measure of similarity. The identities were checked for all 16 states.

The signature projection score S = ‖v‖ cos θ [13,14] differs from α only by the fixed norm of the reference. Both scores therefore order states in the same way. Their dependence on amplitude is intentional, but it matters when a projection is interpreted as directional resemblance.

**Figure 2:**
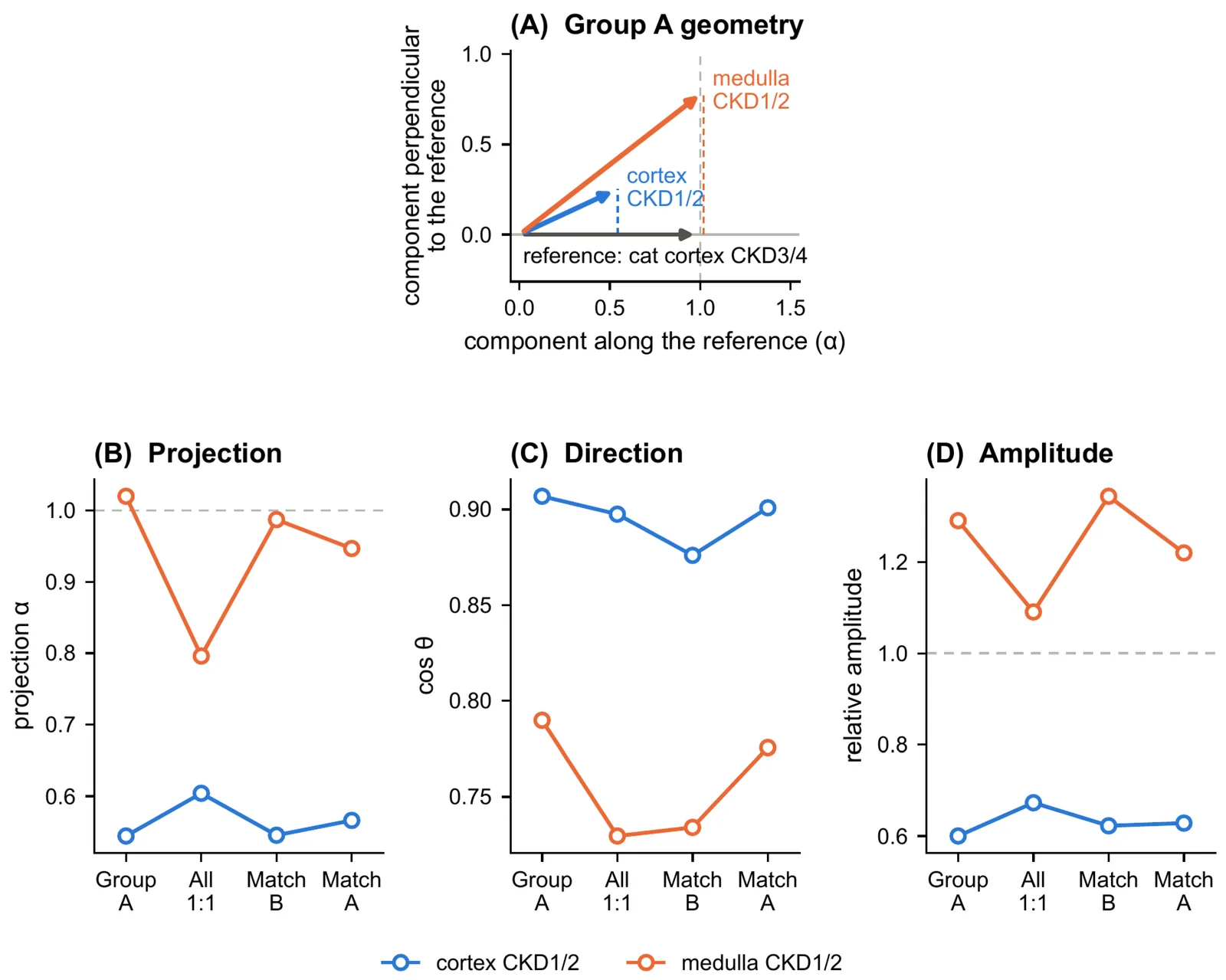
Projection and cosine alignment of the two feline CKD1/2 states. (**A**) Geometry of the Group A point estimates relative to feline cortical CKD3/4, whose norm is set to one. Arrow coordinates use α and ν to recover the component perpendicular to the reference. (**B**–**D**) Projection α, cosine alignment cos θ and relative amplitude ν = ‖v‖/‖a‖ in the four gene spaces, where v is the state vector and a is the reference. The horizontal axis of panels (**B**–**D**) is abbreviated: Group A, all one-to-one orthologues (All 1:1), matched Group B (Match B) and matched Group A (Match A), in the order of Table 1. The two states are identified by the shared legend beneath the panels; in panel (**A**), they are labeled directly. The axis label of panel (**D**) gives the quantity in words to avoid confusion between ν and v. Each panel uses the same two states and the corresponding cortical CKD3/4 reference. Point estimates are shown without uncertainty bars because animal-level resampling was run in Group A only; there the opposite ordering of projection and cosine held in 76.0% of replicates (Section 2.4). Values are read from the projection table of each gene space.

The feline CKD1/2 states illustrate the distinction (Figure 2). Relative to cortical CKD3/4, medullary CKD1/2 had lower cosine alignment than cortical CKD1/2 (0.790 versus 0.907), but a higher projection (1.020 versus 0.544), because its relative amplitude was greater (1.291 versus 0.600). The opposite ordering occurred in all four gene spaces (Table 1). Pod-TRECK day 14 provided a cross-species example: its projection exceeded that of feline cortical CKD1/2 (0.740 versus 0.544), despite lower cosine alignment (0.498 versus 0.907). The condition is exact: for two states with positive alignment, α orders the worse-aligned state first whenever ν_1_/ν_2_ > cos θ_2_/cos θ_1_, that is, whenever the ratio of amplitudes exceeds the reciprocal ratio of alignments. Here that is 1.291/0.600 = 2.15 against 0.907/0.790 = 1.15. A larger response is therefore not sufficient on its own, but any score of the form ‖v‖ cos θ inverts the ordering as soon as the inequality holds. These orderings describe different components of the response and do not rank biological fidelity.

In 2000 animal-level bootstrap replicates that preserved shared controls and cortical–medullary pairing, medullary CKD1/2 retained the larger α in 93.7% of replicates and the lower cosine in 82.3%; both orderings occurred together in 76.0%. The empirical ordering was therefore sensitive to which animals were sampled, even though the algebraic possibility is exact. The resampling included the early CKD groups, the late CKD reference group and their controls (Section 4.11).

The medullary CKD1/2 estimate exceeded α = 1 only in Group A, where it did so under all nine offset and filter settings but in only 46.8% of animal-level resamples. The resampled median was 0.977, with a central 95% range of 0.506–1.397. A projection greater than one is mathematically permitted; it does not mean that a state resembles the reference more closely than the reference resembles itself.

Across the twelve mouse states, whole-panel cosine alignment with the feline reference ranged from 0.19 to 0.55. Projection peaks partly followed response amplitude: Pod-TRECK day 14 had the largest relative amplitude (1.484), while its cosine was similar to day 21 (0.498 versus 0.480). Amplitude can reflect platform dynamic range as well as biological response size, which is why we report it separately from cosine.

Cosine removes a uniform positive scaling factor but remains sensitive to other platform and preprocessing effects. Repeating the decomposition with the Group A union set (2964 genes; 2643 complete) changed estimates only in the third decimal place and did not change the state ordering.

### 2.5. Shared Controls and the Limits of Reliability Benchmarks

The 16 states generated 120 pairwise comparisons, classified as within-dataset, same-species/different-dataset or cross-species (Table 2). Of these, 33 shared control samples. All pairwise estimates are dependent because states and animals are reused.

**Figure 3:**
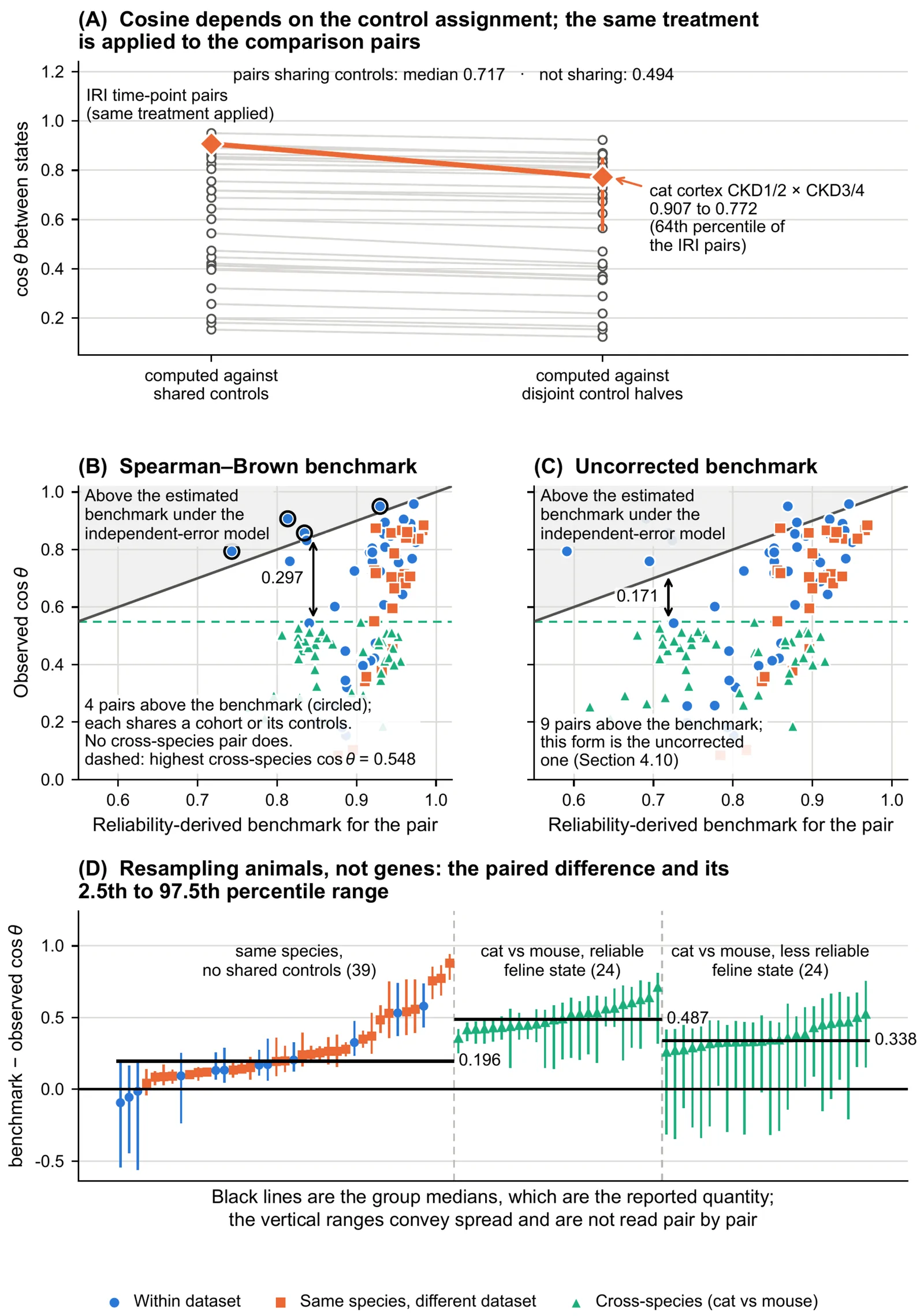
Control assignment and descriptive reliability benchmarks. (**A**) Cosine similarity between feline cortical CKD1/2 and CKD3/4 computed against shared controls and against disjoint control halves, with the same recomputation applied to the 28 IRI pairs used for comparison. The split-control point is the median; the vertical line spans the full range over the 20 assignments of the six controls, not a confidence interval. Under the same procedure, this pair lay at the 64th percentile of 28 IRI pairs. (**B**,**C**) Observed cosine against the Spearman–Brown-corrected and uncorrected benchmarks for all 120 pairs, drawn from the per-pair result table. The diagonal marks equality, not a hypothesis-test threshold or a strict bound. (**D**) For each pair, the 2.5th to 97.5th percentile range of benchmark minus observed cosine over the animal-level resamples, with the median of each group marked by a horizontal line. The groups contain 39 same-species pairs without shared control samples and two sets of 24 cat–mouse pairs, divided by the feline state’s split-half reliability. These are distributions across dependent pairs, not confidence intervals. The grouping variable also enters the benchmark mathematically. Benchmark-gap percentile ranges were poorly calibrated in simulation and are not used to identify individual pairs with a non-zero gap.

In a direct control-splitting check, the cosine between feline cortical CKD1/2 and CKD3/4 fell from 0.907 with shared controls to a median of 0.772 with disjoint control halves (full range over the 20 assignments of 0.553–0.850; Figure 3A). The feline pair lay at the 64th percentile of 28 IRI pairs subjected to the same procedure. Splitting controls adds independent sampling noise as well as removing shared error; this check demonstrates sensitivity to control assignment rather than estimating a bias-free similarity.

Among the 75 cross-dataset pairs, the 27 Pod-TRECK–IRI pairs had a median cosine of 0.707; the 48 cat–mouse pairs had a median of 0.448 and a maximum of 0.548. Twenty of the 27 same-species pairs exceeded that cross-species maximum. The AUC distinguishing these pair classes was 0.7909. This describes separation between the sampled datasets, not a species effect. We do not report a species-label permutation *p* value because states from one dataset are not exchangeable across species labels.

We constructed pairwise reliability benchmarks from split-half RNA estimates, with and without Spearman–Brown correction (Section 4.10). All 48 cross-species point estimates fell below both benchmarks (Table 2; Figure 3B,C). These differences are descriptive: the benchmarks are estimated quantities, not strict upper bounds or calibrated decision thresholds.

The small-sample bootstrap procedure did not provide reliable interval estimates for benchmark gaps. Average coverage in the examined simulations was 9.4% for the uncorrected gap and 49.0% for the corrected gap; for the latter, the lower percentile exceeded zero in 38–76% of datasets with a true gap of zero. We therefore make no pairwise claim that sampling noise has been ruled out. Detailed simulation, undefined-resample and group-summary results are reported in the Supplementary Notes and Figure S4. This calibration failure concerns the procedure that re-estimates split-half reliability; it does not directly establish the behavior of the separate centering-AUC resampling procedure.

Pairs involving the more reliable feline states had larger median benchmark gaps (Figure 3D). Reliability itself enters the benchmark, however, and the 24 pairs in each feline reliability group share only two feline states. The ordering is therefore neither independent replication nor evidence for a biological deficit. Same-species pairs also fell below their benchmarks.

Shared control samples were present in 33 pairs (median cosine 0.717) and absent in 87 (median 0.494). These pair classes differ in composition as well as control sharing.

### 2.6. Exploratory Time-Course and Human Comparisons

Pairwise cosine dissimilarity among mouse states was associated with elapsed time, with Mantel statistics of 0.599, 0.637, 0.646 and 0.613 across the four gene spaces. Alignment with the fixed feline reference was less precise and more sensitive to gene selection. In the human comparison, the apparent feline–mouse ordering reversed after matching genes on sequence identity. These exploratory results do not establish a preferred time point or species. Full estimates and their limitations are provided in the Supplementary Notes, Table S8 and Figure S5.

## 3. Discussion

### 3.1. Centering Changes the Feature of the Response Being Compared

The principal finding is a marked change in pathway-level comparison after subtracting each state’s mean response across genes. With the same Group A genes and pathways, the pair-class AUC changed from 0.868 to 0.511. The direction of this contrast persisted in most animal resamples and across the nine tested offset and filtering settings. Its size was less stable, and the centered score was not consistently near chance. The evidence supports sensitivity to centering within these cohorts, rather than a fixed property of feline or mouse kidney disease, and the state-wide level that carries the contrast may be a dataset-specific technical feature rather than a species-specific biological one.

Centering removes an average defined by the selected gene universe. Uncentered pathway means retain that component; centered means describe relative departures from it. Dividing a state by its standard deviation does not change cosine, so the distinction comes from subtraction of the mean. At the gene level, centering reduced AUC only to 0.759; averaging those centered values produced the larger reduction to 0.511. Both operations therefore matter. The retained mean cannot be interpreted as an absolute change in total RNA per cell, because the input consists of normalized bulk profiles from different platforms.

A higher AUC is not a biological validation target. Here it means stronger separation of same-species and cross-species pairs, which are also separated by dataset. If the question concerns deviations from an overall response, centering may be appropriate. If the overall response is part of the intended comparison, removing it changes the target. Reporting both representations makes that choice visible. This finding applies to arithmetic pathway means compared by cosine. Scores built from within-state ranks are unchanged by centering, and none of the rank-based methods separated the pair classes (Section 2.1, Table S4). The choice we describe therefore arises where pathway scores are arithmetic means of values that keep each state’s overall level, and it does not establish that conclusions from other pathway analyses, including the COVID-19 comparison in [26], would change.

### 3.2. Biological Implications of the Two Representations

Pathway-dependent model agreement is already recognized in congruence analysis [10], malaria [27], Alzheimer’s disease [28] and cross-species translation studies [15]. Our contribution is to examine how score construction alters that agreement within one kidney-disease panel. The two representations answer different questions about the same response. An uncentered pathway mean combines two components: the change in that pathway’s genes and the state’s overall level of change across all measured genes, which every pathway of that state shares. A centered pathway mean retains only the first, expressed relative to the state’s average gene. When a model is judged by a pre-specified fibrosis pathway, for example, the uncentered score is higher for any state whose response is shifted upward across the genome, even if its fibrosis genes move no more than its other genes, whereas the centered score asks whether fibrosis is engaged selectively relative to the rest of the response. The first is a question about the absolute size of the fibrotic response, and the second about its selectivity; they are different questions, and a comparison should state which one it asks.

Which representation is biologically informative depends on what the retained level represents, and that is where the present panel cannot help. The state-wide mean could reflect disease biology, such as a broad shift in transcription or a change in the cellular composition of the sampled tissue, but it is also the component most exposed to platform, normalization and expression-unit differences between datasets. Here all four feline states come from one cohort and one deposited dataset, and all sit below every mouse state on this single number. Attributing the retained level to species biology would require a design in which the compared groups share processing, are contrasted within one dataset, or are tied to an external measurement of the same quantity; none of these applies here.

For methods that score each state from the ranks of its own genes, such as ssGSEA and singscore, the choice does not arise, because the scores are unchanged by centering; it has been made in selecting the method. For the arithmetic pathway means, which the PAGE statistic [29] already takes in centered form, three checks make the situation visible in a given dataset, and all three were carried out here: tabulating the state-wide mean of the response and examining whether it separates the groups to be compared; comparing the score matrices obtained with and without centering, to detect an implicit centering in the scoring method; and reporting both representations side by side, as in Table 1, so that a small difference shows that a conclusion does not turn on the choice and a large one identifies the claims that do.

What follows for model selection is limited. The focal-adhesion examples show that modest whole-panel alignment can coexist with stronger local alignment, but they were selected from a large screen and require testing in prespecified pathways and independent cohorts before they can support a model-selection claim. These data do not contain the material for deciding which representation is biologically more valid: species and dataset are confounded, and neither representation was tested against an external criterion such as prediction of a withheld outcome. The analysis establishes where the two representations diverge and by how much, not which of them is correct, and it does not rank any model.

### 3.3. Pathway Membership, Gene Universe and Measurement Uncertainty

The 422 sets are an overlapping representation of the same genes, more than half of them from Reactome. Analyzing each collection separately, and removing redundant sets, left the contrast unchanged or slightly larger (Section 2.2, Table S5), so it does not depend on that representation in any direction we could detect. The two random-set designs of Section 2.1 separate the averaging operation from the architecture of the collection: size-matched random sets reproduce the contrast in direction at about half the observed size, and sets that also retain the overlap structure of the real collection occasionally reproduce it entirely. Much of what might be attributed to pathway membership is therefore carried by the size and overlap architecture of the collection, and these analyses do not identify which features of that architecture are responsible.

The gene universe is another part of the comparison. Proteome detection selected a relatively highly expressed subset but did not validate RNA measurement reliability, which was estimated from RNA replicates. Expression matching changed the retained genes and cannot isolate a causal expression effect. The centered aggregate score varied with gene selection, whereas the centered-versus-uncentered contrast held in all four spaces (Table 1, Figure 1D). Mapping and precision checks likewise address particular subsets and cannot exclude all technical explanations (Supplementary Notes).

Projection and cosine can favor different states because projection intentionally retains response amplitude. The opposite ordering of the feline CKD1/2 states occurred in all four gene spaces, but in only 76.0% of Group A animal resamples: the algebraic relationship is exact, the empirical ranking uncertain. Reporting amplitude and cosine alongside projection preserves its intended meaning without treating it as a directional-similarity score. None of these quantities was validated against treatment response.

Reliability-derived comparisons are established in congruence analysis and representational similarity analysis [10,30,31]. In these small groups, however, the benchmark and the observed cosine were both uncertain, and re-estimating them in the same animal resample preserved their covariance without producing calibrated benchmark-gap ranges; the simulation result rules out inferential use of those ranges in this study. A larger gap in the higher-reliability group is also partly built into the benchmark and is not independent evidence of biological divergence.

Practical reporting should therefore distinguish four decisions: the gene universe, the centering step, whether amplitude is retained, and which uncertainty the resampling addresses. Gene resampling, animal resampling and random pathway membership answer different questions, and shared control samples and shared animals across tissues must be identified; the 83-pair subset of Section 2.1 addresses the latter dependence more strictly and leaves the contrast essentially unchanged.

### 3.4. Scope and Remaining Limitations

The feline cohort and the two mouse datasets differ in species, disease origin, platform and sampling. Any difference between the pair classes may therefore reflect dataset-specific technical variation—normalization, platform dynamic range, expression units or tissue sampling—rather than species-specific biology, and the state-wide mean through which the centering contrast operates is the component most exposed to such variation. The 120 pairs are not independent observations, and species labels are not exchangeable between states drawn from different datasets, so we do not permute species labels and report the pair-class AUCs descriptively rather than against a permutation null (Section 4.9 and Section 4.12). State-level permutation is used only for the exploratory Mantel analyses of Section 4.9. The intervals we give for gene-bootstrap quantities reflect uncertainty arising from the choice of genes alone and do not contain the uncertainty arising from the choice of animals, which the animal-level resampling of Section 4.11 addresses separately. Neither procedure can compensate for the small number of independent animals, so all of these ranges describe the variability of a statistic under resampling rather than the precision with which a population quantity has been estimated. Bulk tissue composition, age, sex and disease duration were not separated from model differences. An independent kidney-disease cohort with comparable sampling is needed to assess whether the centering effect extends beyond this panel.

The additional time-course and human comparisons illustrate limits on broader interpretation. Temporal organization among mouse states does not imply progression toward a fixed feline profile, and the uncertain reference-alignment estimates do not identify a preferred model time point. The human comparison changed with sequence-identity matching, which alters gene membership as well as conservation. It cannot support a stable species ranking or identify mapping error as the cause. The protein-layer comparison was drawn from available cohort profiles and did not independently replicate the RNA projection reversal.

Most mouse disease groups contained only three animals. Unequal split sizes, repeated animals in bootstrap samples and undefined reliability estimates constrain the benchmark analysis. Pathway overlap and post hoc selection constrain pathway-specific claims. Reproducibility also requires the source proteomic detection tables, exact orthologue and pathway releases, and the deterministic matching list. Finally, similarity to a disease transcriptome was not tested as a predictor of therapeutic translation. That question requires intervention outcomes and a validation design suited to cross-species prediction [15,32].

## 4. Materials and Methods

Notation: gene g, state s, sample i; ctrl(s) is the control group assigned to state s.

### 4.1. Disease States

A state was defined as a disease group contrasted with its assigned control group within one dataset and measurement layer. The 16 transcriptomic states comprised four feline cortical and medullary CKD groups [33], Pod-TRECK at days 5, 14 and 21 [34], and nine IRI time points from 2 h to 12 months [35]. The IRI expression matrix is the single supplementary file of GSE98622, GSE98622_mouse-iri-master.xlsx (43,309 genes × 49 samples, downloaded 26 August 2026, SHA-256 f24d20bc3dcd36115c7273a0303d788db630a5190cf92840e07dc74cce30e58a). Its values are FPKM on the source study’s own account: it states that it quantified fragments per kilobase of transcript per million mapped reads from 43,309 genes and that the full table containing those values for all samples was deposited under this accession [35]. The file we used has 43,309 rows, consistent with that description. The values were used as deposited and transformed by log2(FPKM + 1). Proteomic detection defined Group A; it did not provide the RNA reliability estimates. A supporting within-cohort protein comparison is summarized in the Supplementary Materials. Table 3 gives the control assignment.

### 4.2. Gene Identifier Resolution Across Species

Cat-to-human and mouse-to-human correspondences were obtained from Ensembl BioMart (Ensembl release 115; EMBL-EBI, Hinxton, UK) [36] tables restricted to one-to-one orthologues. Profiles were represented by upper-case human gene symbols. When a source symbol had several rows, the highest orthology-confidence row was retained, with sequence identity used to break ties. Genes absent from the mapping table were matched by upper-case symbol, and mapping provenance was recorded. Rows carrying no gene symbol, recorded as ‘-’ in the two Pod-TRECK tables, were excluded; entries such as GT(ROSA)26SOR are genuine and were retained. Duplicate human symbols were resolved by retaining the first occurrence in the original index order. That rule depends on the order of the input rows. It applies to 26 of the 2016 genes analyzed (1.3%), for which two or more source genes map to the same human symbol in at least one state. Shuffling the input order 100 times and rebuilding the whole analysis each time left the gene count unchanged and moved the gene-level AUC by at most 0.0005, the uncentered aggregate AUC by at most 0.0011, the centered aggregate AUC by at most 0.0048 and the difference between them by at most 0.0043 (0.356 against a range of 0.356 to 0.361). The mean response separated the feline from the mouse states in all 100 orders. The strict orthologue-only sensitivity analysis excluded symbol-only matches.

The main analysis is carried by orthologue-derived genes, since Group A passes both proteome detection and orthologue assignment. The contribution of symbol-matched genes is quantified separately in Supplementary Note S3 and in the sensitivity analysis of Table S9.

### 4.3. Expression Filtering

**Pod-TRECK (FPKM)**. Genes were retained when the group-mean FPKM reached the threshold in at least one group:

$$\underset{k\in \mathrm{groups}}{\max}\left(\frac{1}{\left|k\right|}\sum_{i\in k}{\mathrm{FPKM}}_{g,i}\right)\;\geq \;\theta ,\;\;\theta =1.0$$

**IRI (GSE98622, FPKM).** Within the samples entering a given contrast, max*_i_*log_2_(*x_g_*_,*i*_ + 1) > 1.

The feline data were deposited on a log2 scale. Rows with zero variance were removed, but no additional abundance threshold was applied in the primary analysis. Group A genes had median control-expression percentile ranks of 0.857 in the feline data and 0.810 and 0.828 in the two mouse datasets. Excluding genes below the feline 25th percentile increased the four feline split-half reliabilities by 0.035, 0.008, 0.006 and 0.013. This check does not establish how the benchmark gap changes, because filtering can alter both the benchmark and the observed cosine.

The filter and mapping sensitivities were assessed jointly in Table S9. Removing the expression filter reduced Spearman correlation by 0.067 on average across the factorial settings; symbol matching increased it by 0.012. These averaged contrasts do not identify the source of every individual variant difference, particularly if the settings interact.

### 4.4. Definition of Δ

Each state’s response is the group-mean difference against its own control group on a log2 scale:

$$\Delta_{g,s}\;=\;\frac{1}{\left|s\right|}\sum_{i\in s}y_{g,i}\;-\;\frac{1}{\left|\mathrm{ctrl}\left(s\right)\right|}\sum_{i\in \mathrm{ctrl}\left(s\right)}y_{g,i}$$

Here y denotes log2-scale abundance: deposited log2 values for feline RNA, log2(FPKM + 1) for Pod-TRECK and log2(FPKM + 1) for IRI. This difference in mean log2 values is not the log2 ratio of mean abundances. The offset has different effects in FPKM and count units, particularly near the detection threshold, and can change the direction as well as the size of a response. We therefore use the term difference in mean log2 expression. Table S6 examines selected offset and filtering choices. Where gene-level differential-expression statistics were required, moderated t statistics [37] and Benjamini–Hochberg adjustment [38] were used; these gene-level procedures do not calibrate the pairwise similarity or pathway comparisons.

We did not use the alternative in which each gene is standardized across samples before the difference is taken,

$$z_{g,i}=\frac{\left(y_{g,i}-{\bar{y}}_{g,\mathrm{ctrl}}\right)-{\mathrm{mean}}_{i}\left(\cdot \right)}{{\mathrm{sd}}_{i}\left(\cdot \right)},\;\;\Delta_{g,s}^{z}={\bar{z}}_{g,s}-{\bar{z}}_{g,\mathrm{ctrl}}$$

This alternative produces standardized effect sizes and can reorder genes. Its vector norm is mathematically valid, but measures the size of a standardized response rather than the size of expression changes. Table S9 reports its effect on the selected cross-species correlation.

Three alternative definitions of Δ were carried through the whole 16-state analysis as sensitivity analyses, with the main analysis left at the difference in mean log2 expression (Section 2.1, Table S3). Per-gene standardization divides each gene’s Δ, after subtracting its mean over the sixteen states, by its standard deviation over those states (one degree of freedom). Ranking within a state replaces each state’s column by the ranks of its genes, mapped to (0, 1) as (rank − 0.5)/2016, with ties broken by a stable sort. Quantile normalization sorts each state’s column, averages the sorted columns across the sixteen states to form a reference distribution, and writes that reference back in the order of the original ranks. The last two give every state the same marginal distribution, so their state-wide means are equal by construction; this is a property of the operation and not an independent observation.

### 4.5. Control Assignment

Cortical and medullary feline samples were paired by animal identifier where both tissues were available. The eight IRI states from 2 h to 28 d shared young sham controls, and the three Pod-TRECK states shared uninduced controls. Shared control samples and shared animals across tissues were recorded separately. The 87-pair comparison excluded shared control samples; the 83-pair sensitivity check additionally excluded the four feline cortical–medullary pairs sharing control animals.

The 12-month IRI state was compared with age-matched SHAM12m samples. The 6-month time point, assayed on a second platform, was excluded. These assignments avoid directly contrasting old injured animals with young sham, but do not remove all age-related differences among the states.

### 4.6. Definition of Group A

Proteomic detection was defined separately in each proteome, and the two source tables record an unquantified sample differently. In the Pod-TRECK quantification table (Supplementary Data S1) a protein that was not quantified in a sample is recorded as 0 rather than left blank: the table has no blank cells among its 114,516 sample entries and 4465 zero entries. A zero was therefore read as not quantified, and a protein was called detected when it had a positive value in at least 5 of the 9 samples, that is, in at least half of them. The table has 12,724 data rows, of which 90 carry no gene symbol and are excluded as described in Section 4.2, leaving 12,634 symbols; 12,308 of them met the criterion. In the feline cortical and medullary proteomes an unquantified sample is left blank, and the few exact zeros that do appear (5 in cortex and 4 in medulla) were kept as measured values; a protein was called detected when it had a value in at least 12 of the 23 cortical samples and in at least 10 of the 19 medullary samples, again at least half, giving 2937 of 2967 and 2661 of 2698. The resulting symbols were mapped to the human symbol space and intersected. Group A was based on detection in the feline and mouse proteomes, without selection for disease-response direction. The feline proteomes were from the cohort in [33] and are deposited in ProteomeXchange under PXD066590. The Pod-TRECK proteome was generated in the companion study [39] and is separate from the RNA accession GSE299326; the quantification table used here is the one underlying Supplementary Table S1 of that paper.

Group A_∩_ = (*P*_cat,cortex_ ∩ *P*_cat,medulla_) ∩ *P*_mouse_  Group A_∪_ = (*P*_cat,cortex_ ∪ *P*_cat,medulla_) ∩ *P*_mouse_

That quantification table is provided as Supplementary Data S1, as received and without further processing; it supplies the mouse side of the selection only. The feline side comes from the cortical and medullary proteome sheets of the published supplementary data of [33] (42003_2025_9164_MOESM3_ESM.xlsx, downloaded 26 August 2026, SHA-256 0037263600ec06f688a453ddbc2732e754c9034df7b4486713bfc76663ebf136), which the publisher distributes openly, and the human symbol space from the Ensembl BioMart [36] one-to-one orthologue tables retrieved on 26 August 2026 when the current release was 115. Together these reproduce the selection exactly, 2240 genes in Group A, of which 2016 have finite values in all sixteen states, and the verification script of Section 4.18 performs that recomputation from the attached file. The resulting calls are listed per gene in Table S10.

Group B is every gene in the Δ matrix that is not in Group A. The main analysis uses Group A_∩_ (2240 genes; 2016 with finite values in all 16 states); Group A_∪_ (2964; 2643) serves as a sensitivity analysis and changed no conclusion.

### 4.7. Projection and Its Decomposition

The normalized projection *α*, cosine alignment cos*θ*, relative amplitude *ν* and normalized residual *R*_⊥_ were computed using the definitions and identities in Section 2.4. Unless otherwise stated, the feline cortical CKD3/4 response was used as reference vector *a*, and each state’s response was represented by vector *v*. The projection combines relative amplitude and directional alignment, whereas the normalized residual depends only on cosine magnitude and does not retain its sign. The identities were used as implementation checks; they do not validate a biological interpretation of the scores.

The signature projection score *S* is the scalar projection of the state vector onto the direction of the reference vector [13]. The normalized projection *α* used here is this score divided by the fixed Euclidean norm of the reference vector. Because that norm does not vary between states, *α* and *S* order states identically. Both scores retain response amplitude as well as directional alignment. The patent [14] provides a later formal statement of the same construction.

All quantities were computed on the genes of the chosen set that are finite in every state. If the gene set differed between states, a difference in the number of missing values could appear as a difference in the vector norm.

### 4.8. Pairwise Comparison Without a Fixed Reference

Every pair of the 16 states was compared by cos*θ* and classified as within-dataset, same-species/different-dataset, or cross-species.

Shared-control sensitivity was assessed by dividing controls into disjoint halves A and B and recomputing the two contrasts separately:

$$\Delta_{g,s_{1}}^{\left(A\right)}={\bar{y}}_{g,s_{1}}-{\bar{y}}_{g,A},\;\;\Delta_{g,s_{2}}^{\left(B\right)}={\bar{y}}_{g,s_{2}}-{\bar{y}}_{g,B},\;\;A\cap B=\emptyset$$

We evaluated all 20 assignments of the six controls to two groups of three and used the median cosine. This recomputation was applied only to the two control groups of six animals, the feline cortical controls and the young sham of the IRI series, giving the single feline cortical pair and the 28 IRI pairs; the three Pod-TRECK pairs were excluded because a control group of three cannot be divided into two halves of equal size. Halving the control group adds independent sampling noise and lowers the expected cosine. We therefore applied the same procedure to the comparison set.

### 4.9. Mantel and Partial Mantel Tests

Pairwise associations were assessed with Mantel statistics [40]. We used rank dissimilarity (one minus Spearman correlation), cosine dissimilarity (one minus cosine) and amplitude dissimilarity (the absolute log2 ratio of vector norms). The time predictor was the absolute difference in log10 elapsed hours among the twelve mouse states. Onset compartment and model identity were coded zero for matching states and one otherwise. The statistic was the Pearson correlation between strict upper-triangle entries. States were permuted jointly across rows and columns 9999 times. Two-sided *p* values used the absolute statistic and added one to both the exceedance count and the number of permutations. Partial statistics were obtained by regressing each upper triangle on the control matrix and correlating the residuals under the same state-label permutation scheme. This retains the pair structure but still assumes exchangeability of states under the null. Results are exploratory because model structure, time-course sampling and shared controls may restrict that exchangeability.

### 4.10. Reliability Benchmark

For each state, disease and control samples were partitioned into disjoint groups, and cosine similarity was computed between the two resulting Δ vectors over 500 random splits. The median split cosine was used as the half-sample reliability. The Spearman–Brown expression [41,42,43] was used as an approximate extrapolation to full-sample reliability:

$$r_{\mathrm{XX}}=\frac{2\;r_{\mathrm{half}}}{1+r_{\mathrm{half}}},\;\;r_{\mathrm{XX}}:=0\;\;\mathrm{if}\;r_{\mathrm{half}}\leq 0$$

The uncorrected and Spearman–Brown-corrected pairwise benchmarks were defined as follows:

$${\mathrm{benchmark}}_{\mathrm{XY}}^{\mathrm{raw}}=\sqrt{r_{\mathrm{half},X}\;r_{\mathrm{half},Y}};\;{\mathrm{benchmark}}_{\mathrm{XY}}^{\mathrm{SB}}=\sqrt{r_{\mathrm{XX}}^{\mathrm{SB}}\;r_{\mathrm{YY}}^{\mathrm{SB}}}$$

The benchmark is motivated by an attenuation model with independent measurement errors. Its use here involves two approximations: the observations are uncentered cosines rather than centered Pearson correlations, and split-half reliability is estimated from fewer samples than the full-sample similarity. These distinctions are relevant to comparisons with congruence analysis and representational similarity analysis [10,30,31]. The simulation varied sample size, underlying cosine and reliability, including the observed split-half range of 0.568–0.986 and common mean shifts of 0–0.44 within-state standard deviations. The median empirical shift was 0.257. Figure S4 contrasts centered matched-size comparisons, centered and uncentered half-sample benchmarks, and the corrected uncentered benchmark.

In the simulation, half-sample benchmarks were exceeded by full-sample similarities at high underlying alignment, as a step in reliability rather than a graded rate: at a true cosine of 0.9, both the centered and the uncentered half-sample benchmarks were exceeded in 97–100% of replicates at every simulated reliability up to 0.816 and in no replicate at 0.865 or above, in both sample-size designs. Spearman–Brown correction reduced the sample-size mismatch: within the observed-reliability conditions, non-identical responses did not exceed the corrected benchmark, whereas identical responses exceeded it in 59.4% of replicates (range 57.0–62.0%) under the feline-like seven-versus-six design and in 98.0% (96.3–99.7%) under the mouse-like three-versus-six design, with no consistent trend across the reliability band (Figure S4; Supplementary Note S1). These simulation-specific results do not make the estimated benchmark a strict upper bound or establish calibration of a test. The corrected benchmark is the primary displayed form; the uncorrected form is a sensitivity comparison. Calibration of the benchmark-gap resampling procedure was examined separately in Section 4.11.

The uncorrected benchmark is numerically lower and therefore gives a smaller gap for a fixed observed cosine. That ordering alone does not establish conservative inference, because both terms are estimated and the benchmark targets a different sample size. Table S11 reports the observed cosine, both benchmarks and the observed-to-corrected-benchmark ratio as descriptive quantities. The ratio can exceed one and is not a bounded cosine estimate.

Spearman–Brown extrapolation assumes comparable halves. For groups of three, the equal-half assumption cannot be satisfied if all animals are used, so the extrapolation depends on the partitioning convention. The convention used here splits a randomly permuted group into its first floor (*n*/2) members and the remainder, so a group of three is divided one against two and no animal is dropped; a group of six is divided three against three. Table S12 gives the recorded half-sample counts, in which the tabulated half size is that of the smaller half. Sample-size checks were possible only for feline states and do not establish calibration for the mouse groups of three.

### 4.11. Animal Resampling and Benchmark Gap Calibration

Most mouse disease groups contained three animals. We therefore distinguished sensitivity to gene selection from sensitivity to animal sampling, and examined the calibration of resampling ranges for reliability-benchmark gaps. Pathway examples were treated as exploratory because the sets overlap and because selecting extreme values introduces further uncertainty.

Gene bootstrapping [44] assesses sensitivity to the selected genes, conditional on the observed animals; it does not estimate animal-sampling uncertainty, and resampling of either kind cannot compensate for the small number of independent animals. To examine animal sampling, both the observed similarity and its estimated benchmark were recalculated within the same animal resample.

Animals were resampled with replacement 1000 times for each pairwise benchmark analysis. The sampling unit was the animal identifier. Disease and control groups were combined into pools when their identifier sets overlapped, and one draw of the original pool size was made from each union. Each group then retained the sampled identifiers belonging to it, with multiplicity. Identical control groups received identical resampled controls. Partial cortical–medullary overlap was also retained: early CKD groups of eight and seven shared six animals, late CKD groups of seven and five shared five, and the control tissues shared six. This pooling allows group sizes to vary across replicates. Disjoint pools were sampled independently.

Within each replicate, the response vectors and observed cosine were recalculated. Split-half reliability was then estimated from 50 further partitions of the distinct animal identifiers present, with no identifier placed in both halves. Both corrected and uncorrected benchmarks were formed from these estimates. Re-estimating both terms within the same resample preserves their covariance, but does not by itself guarantee a valid confidence interval.

Replicates with an empty group or fewer than two distinct animals in a group had no defined benchmark. They were neither redrawn nor imputed. The proportion excluded was recorded, and benchmark-gap summaries were conditional on the defined replicates. Drawing three times from three animals gives only one distinct animal with probability 1/9. Across the cross-species analyses, undefined proportions ranged from 0.098 to 0.235.

For each defined replicate, the gap was benchmark minus observed cosine. We summarized its median and empirical 2.5th and 97.5th percentiles, using linear interpolation between order statistics. These summaries describe the resampled statistic. They are not treated as calibrated confidence intervals, and neither percentile exclusion of zero nor a conditional bootstrap tail proportion is used as a hypothesis test.

Simulation calibration of the benchmark-gap procedure is described in Supplementary Note S1. The resulting percentile ranges had poor coverage and were retained only as descriptive sensitivity summaries. No pairwise inferential decision was based on whether a benchmark-gap range excluded zero.

The centering comparison used 1000 animal-level resamples with the same identifier-pooling scheme across all 16 states. Each draw yielded new Δ vectors, 422 pathway means with and without centering, and both AUCs for the same 39 same-species and 48 cross-species pairs without shared control samples. We report the proportion with uncentered AUC greater than centered AUC and the percentile summaries of each AUC and their difference. No replicate was undefined. This procedure does not require re-estimating split-half reliability, so the benchmark-gap calibration failure does not directly apply to it. Its percentile ranges nevertheless remain resampling summaries, not independently validated confidence intervals. The same procedure was repeated over the 75 cross-dataset pairs of Table S1, which additionally exclude the twelve pairs that are within one dataset as well as within one species, with every other setting unchanged.

A leave-one-animal-out check was also performed by removing each identifier from every group in which it occurred. Replicates leaving fewer than two animals in a group, or an undefined benchmark, were omitted. Under the uncorrected benchmark, at least one deletion removed the positive gap in one of the 48 cross-species pairs. This is a local sensitivity check and does not establish a sign or significance for the other pairs.

### 4.12. Descriptive AUC Comparisons

AUC was defined as the probability that a randomly chosen same-species pair had a higher similarity than a randomly chosen cross-species pair, with ties assigned half weight. The 75-pair analysis comprised 27 cross-dataset mouse–mouse pairs and 48 cat–mouse pairs. The 87-pair analysis comprised 39 same-species pairs without shared control samples and the same 48 cat–mouse pairs. Excluding four additional feline cortical–medullary pairs sharing control animals left 83 pairs. These pairs are dependent because they reuse states and animals. AUC is therefore a descriptive measure of pair-class separation, not a cross-validated classifier performance estimate. No species-label permutation *p* values are reported. Table S1 records the pair definitions.

### 4.13. Pathway-Level Analysis

Gene sets were drawn from Reactome [18], MSigDB Hallmark [21], Gene Ontology Biological Process [19] and KEGG [20]. Sets containing at least 30 Group A genes were retained, yielding 422 sets: 229 Reactome, 118 GO Biological Process, 48 KEGG and 27 Hallmark. Three names occurred in two collections with different membership and were retained separately. Sets were not independent: the median maximum Jaccard overlap of a Reactome set with a KEGG set was 0.467, and 49% reached 0.5. Each pathway-specific reference comparison used only that set’s genes. Adding Reactome changed the median pathway AUC from 0.788 to 0.760 and its rank correlation with size from 0.035 to 0.198.

For each tested set size, 300 random gene sets were drawn from the same gene universe and their central 95% cosine range recorded; these distributions describe gene-set backgrounds, not animal-sampling uncertainty. Two random-set designs were applied at the aggregation step, each with 300 draws from the same seed (Section 2.1). In the first, 422 random gene sets matched to the sizes of the real ones were formed; each state was reduced to the corresponding 422 means with and without centering, and the aggregate AUC was recomputed. Because independent draws do not reproduce the gene overlap between the real sets, the second design retained the 422 real sets and permuted the gene labels of the Δ matrix, preserving set sizes, all pairwise overlaps and the number of sets each gene belongs to, and breaking only the correspondence between gene identity and set membership. The draw-level values of both designs are released with the code. To separate the number of pathway coordinates from the choice of gene space, 163 of the 422 Group A set was also drawn at random 300 times, and the aggregate AUCs were recomputed on that reduced set of coordinates. Individual mouse-state-by-pathway cells were highlighted only if the pathway contained at least 50 genes, the cosine was at least 0.9 and it exceeded the matched random-set 97.5th percentile; this was an exploratory screen, not a multiplicity-adjusted test, and animal resampling of the selected examples did not account for their selection.

Three variations in the pathway set itself were examined and are reported in Table S5, with the gene universe, the response definition and the 87 pairs held at their main values. First, the aggregate AUCs were recomputed within each source collection separately. Second, redundant sets were removed greedily: sets were visited in order of decreasing size and a set was dropped when its Jaccard overlap with an already retained set reached a threshold of 0.5, 0.3 or 0.2. Third, because the 30-gene criterion is applied within each gene space, the four spaces of Table 1 do not contain the same pathways; the 117 sets, identified by collection and name, that qualify in all four were used to recompute the four differences over identical pathway coordinates.

Figure S2B displays 74 qualifying sets from GO Biological Process, KEGG and Hallmark. The 104 Reactome sets with at least 50 Group A genes were omitted from that heatmap for readability. Numerical summaries use all 422 sets unless otherwise stated. The displayed subset should not be interpreted as a non-redundant or independently selected validation set.

For pairwise pathway-specific comparisons, cosine was computed within each pathway for the 87 pairs without shared control samples. For aggregation, each state was represented by its vector of 422 pathway means. The centered version standardized Δ within each state across the common gene universe before taking those means, so that each pathway mean is taken after the state-wide mean has been removed, as in the numerator of the PAGE statistic [29]:

$$z_{g,s}=\frac{\Delta_{g,s}-{\mathrm{mean}}_{g}\left(\Delta_{\cdot ,s}\right)}{{\mathrm{sd}}_{g}\left(\Delta_{\cdot ,s}\right)},\;\;m_{P,s}=\frac{1}{\left|P\right|}\sum_{g\in P}z_{g,s}$$

so that each state becomes a vector of 422 pathway means, *m_P_*_,*s*_, and two states are compared by the cosine between those vectors.

Only the centering in that standardization can change a cosine. Dividing a state’s vector by its own sd*_g_*(Δ_⋅,*s*_) is multiplication by a positive scalar, which cancels in cos*θ*, and it survives the pathway averaging as a common factor of every *m_P_*_,*s*_ for that state, so it cancels there too. Consequently cos(*z*_⋅,*i*_,*z*_⋅,*j*_) equals the Pearson correlation of *Δ*_⋅,*i*_ and Δ_⋅,*j*_, and the aggregated comparison is unchanged if the scaling is omitted; both identities were verified numerically to within 3 × 10^−15^. Section 2.1 therefore reports four conditions—the cosine of Δ, the cosine after centering each state, and the pathway means taken with and without that centering—since the within- and cross-species distributions, not the AUC alone, say in which direction each step moved the classes.

### 4.14. Alternative Pathway Scoring Methods

Four scoring methods were compared with the arithmetic pathway mean over the same 422 sets and the same 87 pairs (Section 2.1, Table S4). Each was applied twice, once to the Δ matrix and once to the same matrix after centering, using the Bioconductor implementations under R 4.6.1 and Bioconductor 3.23: GSVA 2.6.6 for ssGSEA [22], GSVA [23] and PLAGE [25], and singscore 1.32.0 for singscore [24], with GSEABase 1.74.0 reading the gene sets. Cosine similarities and the aggregate AUCs were then computed from the resulting score matrices by the same Python code used elsewhere in this work (Python 3.9.6, NumPy 2.0.2), so no AUC is calculated in R.

The input to every method was the 2016 × 16 matrix of Δ values, used in place of an expression matrix, with the sixteen states in the position of samples. This is not the use these methods were designed for: ssGSEA ranks genes within what it treats as a sample, and the GSVA kernel density estimate is formed across what it treats as samples, here sixteen state-level contrasts rather than individual animals. The comparison therefore shows how each scoring rule responds to centering of this matrix, and the scores are not interpretable as pathway activities of individual animals.

Methods were specified through the GSVA parameter-object interface: ssgseaParam with alpha = 0.25 and normalize = FALSE, gsvaParam with kcdf = ‘Gaussian’, tau = 1, maxDiff = TRUE and absRanking = FALSE, and plageParam, each with minSize = 1 and maxSize = Inf because the sets had already been restricted to at least 30 Group A genes. Singscore was run as rankGenes followed by simpleScore with a single undirected up-set, knownDirection = TRUE and centerScore = TRUE. Three settings that could in principle matter were checked and did not: normalize = TRUE for ssGSEA rescales all scores by one global constant and leaves every cosine unchanged; kcdf = ‘auto’ selects the Gaussian kernel for these continuous values; and the arbitrary sign of each PLAGE singular vector reverses that coordinate for all sixteen states at once, so fixing the signs gave the same AUCs to four decimal places.

The four score matrices were also computed independently in Python, with GSEApy 1.3.1 [45] for ssGSEA and GSVA and our own implementations of singscore and PLAGE, and compared cell by cell against the Bioconductor matrices. ssGSEA and PLAGE agreed to better than one part in 10^9^, and GSVA to 5 × 10^−15^ for the median cell and 5.3 × 10^−3^ at worst. Our singscore implementation differed by at most 0.026 (median 0.0021, cell-level correlation 0.9998) because it rescales the mean rank differently from the package, giving an aggregate AUC of 0.480 against 0.482; we report the package values. All version numbers and the session record are in the repository.

Centering subtracts one constant from every gene of a state and so leaves the within-state ranks unchanged, which was verified for all 2016 genes and all sixteen states. Any score that reads the matrix only through those ranks is therefore unchanged by centering as an algebraic consequence, which is what the score matrices show for ssGSEA and singscore, whose largest absolute difference between the centered and uncentered scores was exactly zero. GSVA and PLAGE read the values themselves and are not invariant, with the largest absolute differences of 0.572 and 1.223; the arithmetic mean shifts by the state mean itself, 0.4261 at most.

### 4.15. Matching on Gene-Level Covariates

Whether a difference in ρ between two gene sets is explained by a covariate was assessed with three estimators. (i) **Decile resampling**: one set is resampled without replacement to the decile composition of the other; where a decile is short the estimator falls back to sampling with replacement, and this is recorded. This estimator was implemented but is not used for the comparison reported, because Group A is nearly absent from the low-expression deciles and the fallback would apply in nine of ten (Supplementary Note S3). (ii) **One-to-one nearest-neighbor matching** on the covariate’s percentile rank, without replacement, caliper 0.02, with the difference’s interval from bootstrapping the matched pairs. (iii) **Decile stratification:** weighting each decile that holds at least 20 genes from both sets by $w_{d}=\min\left(n_{d}^{A},n_{d}^{B}\right)$.

Covariates were the control-group mean expression, the empirical Bayes standard error of Δ, ${\mathrm{se}}_{g}=\sqrt{s_{\mathrm{post},g}^{2}\;\left(1/n_{\mathrm{case}}+1/n_{\mathrm{ctrl}}\right)}$, and the empirical split-half noise of Δ, the standard deviation of $\Delta_{g,b}^{\left(1\right)}-\Delta_{g,b}^{\left(2\right)}$ over *B* = 400 splits. Each covariate was converted to a percentile rank within its dataset before averaging, because the columns are on different scales. The two precision measures agree at Spearman ρ = 0.999.

For the gene-space sensitivity analysis, matching was run once in a deterministic order and the pair list was retained. Group A genes were processed in ascending expression rank and assigned the nearest unused Group B gene within a caliper of 0.02, with gene names breaking ties. This yielded 1632 pairs. The 384 unmatched Group A genes had median expression rank 0.949. The matched Group A and Group B spaces were the two sides of that list. The 1754-pair matching in Supplementary Note S3 was a separate application to genes complete in that selected state comparison and should not be confused with these spaces.

### 4.16. Exploratory Comparison with Human Data

Human tubulointerstitial expression profiles from the European Renal cDNA Bank (GEO accession GSE104954) [46] were used to construct three human states—all CKD, diabetic nephropathy and rapidly progressive glomerulonephritis, each against living-donor tissue—by the same Δ definition of Section 4.4. Feline, mouse and human profiles were placed in the common human symbol space of Section 4.2, and each animal state was compared with each human state by cos*θ*.

We examined sensitivity to sequence identity by one-to-one matching at calipers of 2, 5 and 10 percentage points and by using equally sized random gene subsets. Sequence identity was treated as a conservation covariate, not a measured mapping-error rate. The human disease contrasts overlap and shared living-donor controls. No reliability benchmark or inferential species comparison was based on these profiles.

### 4.17. Robustness to the Choice of Gene Space

The projection decomposition, time associations, pairwise cosine and reliability benchmarks, and pathway-specific versus centered-aggregate comparisons were repeated in three additional spaces: all one-to-one orthologues, matched Group B and matched Group A. The gene list was changed while retaining the stated analysis definitions. The centered-versus-uncentered aggregate contrast was computed in all four spaces and is given in Table 1. The offset and filtering sensitivity analysis is given separately in Table S6.

### 4.18. Reproducibility

The reported random seed was 20260826. Gene-bootstrap analyses used 2000 resamples for the intervals on α, cos θ and ν, and state-bootstrap analyses used 5000 for the interval on the per-state correlation between cosine alignment and log elapsed time. Animal resampling used 2000 draws for the feline projection example and selected focal-adhesion comparisons, and 1000 for benchmark gaps and the centering AUC comparison. Mantel analyses used 9999 permutations. Size-matched random-set analyses used 300 draws, split-half reliability of 500 splits, split-half noise of 400 splits and within-bootstrap reliability of 50 splits. Code and derived inputs are described in the Data Availability Statement. A verification script checks every number reported here against the result file it comes from, asserts the projection identities of Section 2.4 and, for the quantities of Section 2.1, recomputes the centering and aggregation tables and the per-state leading shares from the Δ matrix and the gene-set definitions and compares them cell by cell with the stored tables. It also reads a provenance table recording the script that writes each result file, and every value printed inside a figure is written out as the figure is drawn and checked against the text and the caption. Generative-AI assistance is disclosed in the Acknowledgments.

## 5. Conclusions

Mean-centering before pathway averaging substantially changed the comparison of feline and mouse kidney-disease profiles in this panel. The measurement is sensitive to preprocessing at two separate levels: how the disease-response vector is defined, and whether each state’s mean across genes is removed before pathway averaging. The direction of the effect persisted across most animal resamples, the tested offset and filtering settings, all four gene spaces and the 83-pair subset that excludes pairs sharing animals across tissues; its size varied with the gene space and with which pathways qualified in each. Centered and uncentered means compare different features of a response, and stronger pair-class separation does not establish greater biological validity.

When transcriptomic similarity is used to characterize disease models, the gene universe and centering step should be explicit, projection should be accompanied by its amplitude and cosine components, and resampling summaries should be distinguished from validated uncertainty estimates.

Two limitations bound these conclusions. All results derive from one feline cohort and two mouse datasets, so species cannot be separated from the dataset, and the component that drives the contrast may be technical rather than biological. No independent validation dataset was available: whether the centering effect, and the size of the contrast, generalize to other cohorts, platforms and diseases has not been tested and requires independent datasets with comparable sampling. The data also do not establish predictive value for treatment outcomes.

## Figures and Tables

**Table 2:** Observed cosine similarities and estimated reliability benchmarks by pair class. Counts above a benchmark refer to point estimates, not statistical decisions. Brackets give the minimum in each class. Spearman–Brown correction extrapolates toward full-sample reliability but does not produce a validated upper bound.

Pair Class	*n* Pairs	cos θ Median	cos θ Max	S–B Benchmark, Median [min]	Uncorrected Benchmark, Median [min]	Above S–B, Point Estimate	Above Uncorrected, Point Estimate
Within dataset	45	0.755	0.958	0.920 [0.743]	0.852 [0.591]	4	8
Same species, different dataset	27	0.707	0.884	0.945 [0.875]	0.896 [0.781]	0	1
Cross-species (cat vs. mouse)	48	0.448	0.548	0.862 [0.765]	0.765 [0.620]	0	0

**Table 3:** Control assignment for the sixteen states, with the number of disease and control samples entering each contrast.

State	*n* Disease	Control Group	*n* Control
Feline cortex, CKD1/2	8	cortical control animals	6
Feline cortex, CKD3/4	7	cortical control animals	6
Feline medulla, CKD1/2	7	medullary control animals	6
Feline medulla, CKD3/4	5	medullary control animals	6
Pod-TRECK D5/D14/D21	3 each	uninduced control	3
IRI 2 h, 4 h, 24 h, 48 h, 72 h, 7 d, 14 d, 28 d	3 each	young sham (SHAM4h + SHAM24h)	6
IRI 12 mo	3	age-matched SHAM12m	3

## Data Availability

The transcriptomic datasets are deposited in the Gene Expression Omnibus under GSE303653 (feline CKD), GSE299326 (Pod-TRECK; described in [34]), GSE98622 (murine IRI [35]) and GSE104954 (human tubulointerstitial profiles [46]). Code and derived data are provided at https://github.com/masakiwatanabe-labani/ckd-model-distance (accessed on 12 October 2026). The repository contains the Δ matrix, Group A and Group B gene lists, proteomic detection calls, analysis result tables, the figure scripts and the verification script of Section 4.18. It also carries a provenance table naming the script that writes each result file, and a table mapping each quantity reported here to the file it is read from. Orthologue assignments were obtained from Ensembl BioMart [36] and pathway sets from the resources specified in Section 4.13. The feline proteome is deposited in ProteomeXchange under PXD066590 [33]. The Pod-TRECK proteome is not part of GSE299326; it was generated in the companion study [39], and the quantification table used here is provided as Supplementary Data S1, with the per-gene detection calls in Table S10 and the recomputation described in Section 4.6.

## Supplementary Materials

The following supporting information can be downloaded at https://www.mdpi.com/article/10.3390/ijms27209115/s1.
