# Supplementary material for: Mean-Centering Alters Pathway-Based Transcriptomic Comparisons of Feline and Mouse Kidney Disease
Source: Int J Mol Sci. 2026 Oct 14;27(20):9115. doi: 10.3390/ijms27209115 (PMC13705475; doi:10.3390/ijms27209115)
Supplement: Supplementary file 1 [file ijms-27-09115-s001.zip › Supplementary_Notes.pdf]

Supplementary Materials

Mean-Centering Alters Pathway-Based Transcriptomic Comparisons of Feline and Mouse Kidney Disease

Masaki Watanabe, Takeru Sasaki, Ryuya Nakagawa and Nobuya Sasaki

Supplementary Note S1. Reliability and control sensitivity

Amplitude dissimilarity did not separate the pair classes in the way direction did. The median amplitude dissimilarity, defined as the absolute log2 ratio of the vector norms, was 0.535 for same-species pairs and 0.371 for cross-species pairs. The same-species set includes states with markedly different response sizes, such as IRI at 2 h and 24 h. Directional and amplitude comparisons therefore describe different features of this panel.

All 48 cross-species point estimates were below both benchmarks. The lowest corrected benchmark was 0.765, compared with a maximum observed cosine of 0.548; the smallest pair-specific difference was 0.297. The corresponding uncorrected values were 0.620 and 0.171 (Table 2). These are descriptive differences between estimates, each of which is subject to sampling error.

We examined that error by resampling animals 1,000 times, preserving shared controls and cortical–medullary pairing, and recalculating cosine and benchmark from the same resample. A median of 11.8% of replicates per cross-species pair had an undefined benchmark (range 9.8–23.5%), usually because a resampled group contained fewer than two distinct animals. All benchmark-gap resampling summaries are conditional on the remaining replicates.

For the corrected benchmark, the median pairwise resampling summary was 0.487 (interquartile range 0.437–0.561) among the 24 cat–mouse pairs involving feline cortical CKD3/4 or medullary CKD1/2. It was 0.338 (0.323–0.434) among the 24 involving cortical CKD1/2 or medullary CKD3/4, and 0.196 (0.117–0.360) among the 39 same-species pairs without shared control samples. The corresponding uncorrected summaries were 0.403, 0.215 and 0.119. Figure 3D shows these distributions descriptively.

Same-species pairs also fell below their benchmarks, and their gap distribution overlapped those of the cross-species pairs. The observed ordering of the group summaries should not be read as an established difference between species. Platform effects, tissue composition and annotation differences are not assessed by resampling animals within the same cohorts.

A reliability-versus-sample-size check was possible only for the feline states. At half-sample sizes of two and three, Spearman–Brown predictions differed from observed reliability by −0.035 to +0.088, with a median difference of +0.006. The observed value exceeded the prediction in five of seven comparisons. This limited check does not establish calibration for the mouse states, whose disease groups contained three animals.

Four pairs exceeded their corrected benchmark by up to 0.094. All shared a cohort or control samples. This does not identify the cause: in the simulation, independently measured states with identical underlying responses also frequently exceeded the estimated corrected benchmark. Exceedance alone therefore does not diagnose dependent errors.

Calibration was examined in four representative cat–mouse simulation designs reproducing the group sizes, reliabilities and re-splitting procedure. The target was the median benchmark gap over repeated samples from each fixed design. Average coverage by the nominal central 95% bootstrap range was 9.4% for the uncorrected gap (range 0.8–32.5%) and 49.0% for the corrected gap (12.5–80.8%). The target lay above the upper percentile in 90.6% and 51.0% of simulated datasets, respectively. Resamples contained fewer distinct animals than the original groups, which was associated with lower reliability estimates and downward-shifted gaps. Undefined proportions were 0.109–0.212. In zero-gap simulations, the corrected range's lower percentile exceeded zero in 38–76% of datasets. The uncorrected procedure had no such exceedances in the four simulated designs, but its poor coverage and limited null grid preclude a general one-sided guarantee. We therefore retain all benchmark-gap resampling results as descriptive sensitivity analyses.

Supplementary Note S2. Pathway and projection sensitivity

Whole-panel alignment concealed some high pathway-specific values. Forty-five of the 5,064 mouse-state-by-pathway cells had a cosine of at least 0.9. Among the 2,136 cells from sets with at least 50 genes, three reached

0.9 and exceeded the 97.5th percentile of the size-matched random-set background. All three were in focal adhesion (53 genes): IRI 7 d, 0.935; IRI 72 h, 0.912; and IRI 28 d, 0.902. Their respective random-set thresholds were 0.754, 0.762 and 0.746. In 2,000 animal-level resamples, their medians were 0.925, 0.899 and 0.891, with percentile ranges of 0.872–0.945, 0.818–0.942 and 0.822–0.921. These examples were selected after scanning the pathway results. No multiplicity-adjusted inference was performed, and the resampling ranges do not account for that selection. They illustrate local alignment without establishing a focal-adhesion-specific finding.

The primary pathway set contained a median of 45 genes (interquartile range 33–67), and 244 of 422 sets contained fewer than 50. Each of the 16 states was projected onto the feline cortical CKD3/4 reference within each pathway, giving 6,752 pathway-by-state values. Figure S2B displays 1,184 values from 74 GO Biological Process, KEGG and Hallmark sets. Reactome sets were retained in the numerical summaries but omitted from this display.

The original gene-bootstrap ranges for feline cortical and medullary CKD1/2 cosine alignment were 0.893–0.920 and 0.771–0.808; the corresponding projection ranges were 0.520–0.570 and 0.965–1.079. They describe sensitivity to gene resampling conditional on the observed animals. The animal-level ordering check in Section 2.4 addresses a different source of variation.

Pathway-specific AUC was positively associated with gene count (Spearman correlation 0.198). Low values in oxidative phosphorylation and high values in MAPK-related sets were exploratory summaries and were not tested as individual biological findings. The 5th–95th percentile range of pairwise correlations between pathway rankings was −0.385 to 0.888.

Supplementary Note S3. Orthologue mapping and gene selection

We examined two separate questions (Table S7). First, we asked whether pairwise cosine changed with orthologue sequence identity or confidence. Second, we asked how selecting proteome-detected genes affected a fixed cross-species comparison. The latter contrast, between Group A and the remaining genes in Group B, is not a decomposition of the difference between same-species and cross-species pairs.

Cross-species median cosine across increasing sequence-identity quartiles was 0.479, 0.496, 0.495 and 0.327; the corresponding same-species values were 0.743, 0.700, 0.724 and 0.685. Restriction to 1,550 high-confidence one-to-one orthologues gave medians of 0.462 and 0.709. These checks did not remove the class difference. They do not exclude mapping error: sequence identity reflects evolutionary conservation, and changing the gene subset also changes its functional composition.

Group A and Group B had similar median precision ranks, whether assessed by the empirical Bayes standard error of Δ (0.479 versus 0.493) or split-half noise (0.480 versus 0.492). The two measures were highly correlated (Spearman $\rho = 0.999$). Matching on them gave Group A-minus-Group B differences in cross-species correlation of 0.2403 (gene-bootstrap range 0.1876–0.2914) and 0.2107 (0.1634–0.2593), compared with 0.2104 before matching. Thus, neither matching procedure reduced the difference. Similar estimated errors do not establish equal signal-to-noise ratios, because the underlying effect distributions may differ.

Expression differed more clearly between the sets: median ranks were 0.774 in Group A and 0.428 in Group B. Matching 1,754 gene pairs on expression rank reduced the correlation difference from 0.2104 (gene-bootstrap range 0.1746–0.2456) to 0.1564 (0.1024–0.2127). Both matched groups had median expression rank 0.716. Decile-stratified weighting gave 0.1735, with Group A higher in eight of ten deciles. This attenuation of about one quarter is descriptive; matching changes which genes are compared and does not establish a causal contribution of expression. Decile resampling was not used because sparse Group A representation in low-expression deciles would require replacement sampling in nine of ten deciles.

As a separate procedure check, the difference between orthologue-backed and symbol-matched genes was 0.0046 (gene-bootstrap range −0.0526 to 0.0623). This shows that the procedure need not return a large difference, but does not rule out selection effects in the Group A comparison.

Figure S1 summarizes the orthologue and gene-selection checks of this section. Table S9 reports all eight combinations of mapping method, expression filtering and Δ definition for feline cortical CKD3/4 versus Pod-TRECK day 14. The average effects on Spearman correlation were −0.196 for per-gene standardization, −0.067 for

removing the expression filter and +0.012 for symbol matching instead of orthologue mapping. These are sensitivity contrasts within this analysis, not estimates of general technical bias.

Supplementary Note S4. Exploratory time-course and human comparisons

We next examined whether mouse states close in elapsed time were also close in their response profiles. This pairwise question does not depend on the feline reference.

Across twelve mouse states, the Mantel correlation between rank-based dissimilarity and the difference in log10 elapsed hours was 0.596 (state-permutation $p = 0.0044$). The corresponding onset-compartment statistic was −0.064 ($p = 0.727$), and the partial time statistic controlling for compartment was 0.594 ($p = 0.0021$). Onset compartment was perfectly confounded with model identity in the mouse data, so the compartment result cannot establish whether glomerular and tubular initiation have similar effects. These tests describe exploratory associations within the sampled time courses.

For cosine-based dissimilarity, the time statistic remained positive within IRI alone (0.627; $p = 0.0009$), after removing the 12-month state from the full mouse panel (0.627; $p = 0.0043$), and after removing it from IRI alone (0.737; $p = 0.0005$). These checks reduce, but do not eliminate, concerns about model structure and the extreme time point.

To distinguish changes in direction from changes in response size, we compared rank dissimilarity, cosine dissimilarity and the absolute log2 ratio of response norms (Figure S5; Table S8).

The time association was 0.599 for cosine dissimilarity and 0.312 for amplitude dissimilarity in the full panel. Their ratio was 1.92, but it fell to 1.23 within IRI and to 1.17 within IRI after excluding the 12-month state. The direction–amplitude contrast therefore depends on which states are included. We do not interpret the full-panel ratio as a general property of disease progression.

Alignment to the fixed feline reference gave a different and uncertain result. Its correlation with log elapsed time was 0.074, with a state-bootstrap range of −0.720 to 0.720; the amplitude correlation was −0.070. The broad range does not establish absence of a time trend. Moreover, the alignment coefficient was 0.627 when all one-to-one orthologues were used (Section 2.2). We draw no recommendation about choosing an earlier or later model time point from these reference-anchored estimates.

In an exploratory comparison with human tubulointerstitial profiles from GSE104954, feline states had a higher median cosine than mouse states (0.465 versus 0.390; descriptive AUC 0.8981). The median cat–human and mouse–human orthologue sequence identities differed (89.0% and 92.4%). Matching genes on identity at a two-percentage-point caliper reversed the sign of the difference (−0.058). An equally sized random subset did not show the same behavior. This demonstrates sensitivity to gene selection, not that mapping error caused the original ordering: sequence-identity matching also changes the genes and functions represented. The human contrasts overlap and the animal states share datasets, so we do not attach an inferential p value or use this analysis to rank species by human disease fidelity.

Supplementary Note S5. Supporting protein layer comparison

In the supporting comparison of the available protein and RNA profiles, the reported state-rank correlation between layers was 1.000. Median alignment was 0.650 in the protein layer and 0.824 in RNA. The within-cohort projection reversal was not reproduced in protein. These summaries concern the available measured profiles and are not evidence from an independent cohort.

Supplementary Tables

The tables themselves are supplied as Supplementary_Tables.xlsx, one sheet per table. Their captions are reproduced here.

Table S1. Pair subsets used for descriptive AUC calculations. Species-label permutation results are not included because state labels are not exchangeable across datasets. The 120 pairs are generated from 16 states and are not independent observations.

Table S2. Mean response of each state over the 2,016-gene Group A universe. The mean Δ is the component that every pathway mean of that state shares, and that centering removes before pathway averaging (Section 2.1). SD and median are over the same genes, and the last column gives the proportion of genes with a positive Δ. These are descriptive summaries of one gene universe; they are not estimates of a global expression shift, and a common shift may reflect disease regulation, cellular composition or assay properties.

Table S3. The 16-state analysis under four definitions of the disease-response vector. The main analysis uses the difference of mean log2 expression; the other three are sensitivity analyses. Mean Δ is the average over the 2,016 Group A genes; the range is given separately for the four feline and the twelve mouse states. Ranking and quantile normalization give every state the same marginal distribution, so their mean Δ is identical for all sixteen states by construction and the absence of separation there is not an independent result. AUCs use the 87 state pairs without shared control samples and describe pair-class separation, not classification performance.

Table S4. The centering contrast under five pathway scoring methods, over the same 422 sets and the same 87 state pairs without shared control samples. Pathway mean is the method used in the main analysis. ssGSEA and singscore read the Δ matrix only through the ranks of genes within a state, and their centered and uncentered score matrices are identical, because subtracting one constant from every gene of a state does not change that ranking. GSVA also ranks genes within a state, but it first converts values to a kernel cumulative distribution estimated across the sixteen states, so it is not invariant; PLAGE takes the first right singular vector of the set after standardizing genes across states and is likewise not invariant. All four alternative methods are the Bioconductor implementations named in the second column, run under R 4.6.1 and Bioconductor 3.23; the pathway mean has no counterpart there and is the arithmetic mean used in the main analysis. Implementations, package versions and all arguments are given in Section 4.14. AUCs describe pair-class separation, not classification performance.

Table S5. Dependence of the centering contrast on the composition of the pathway set. All rows use the 87 state pairs without shared control samples and the difference of mean log2 expression as the response, and differ only in which gene sets enter the aggregation. Collection rows take one source collection at a time. Redundancy-reduced rows keep one representative of each group of sets whose pairwise Jaccard overlap reaches the stated threshold. Common-set rows restrict all four gene spaces of Table 1 to the 117 sets that reach 30 genes in every space, so that the four differences are computed over identical pathway coordinates; the last column repeats the difference obtained on each space's own qualifying sets, which is the value given in Table 1. Differences are calculated before rounding and can differ by 0.0001 from subtraction of the displayed AUCs. AUCs describe pair-class separation, not classification performance.

Table S6. Sensitivity of pair-class AUCs and two projection indicators to the log2 offset and group-mean FPKM filter. Each AUC uses the 87 pairs without shared control samples. The centering difference is uncentered minus centered pathway AUC, calculated before rounding; it can differ by 0.001 from subtraction of the displayed AUCs. The ordering indicator records whether medullary CKD1/2 has higher α but lower cosine than cortical CKD1/2. The final column records whether the medullary point estimate exceeds $\alpha = 1$. The table does not contain the individual α, cosine or ν values.

Table S7. Orthologue characteristics and the effect of selecting proteome-detected genes. Block 1 compares pairwise cosine across gene subsets. Block 2 compares Group A and Group B within a fixed cross-species comparison; it does not decompose the same-species versus cross-species difference. Δρ is the Group A-minus-Group B difference in cross-species Spearman correlation.

Table S8. Mantel statistics for elapsed time and onset compartment over the twelve mouse states, with direction and amplitude separated. The elapsed-time predictor is the absolute difference in log10 hours between two states; the row controlling for onset compartment gives partial Mantel statistics.

Table S9. Sensitivity of Spearman correlation between feline cortical CKD3/4 and Pod-TRECK day 14 to mapping, filtering and response definition. Mean log2 difference is the difference between group means after log transformation, not the log2 ratio of group means. Standardized denotes the per-gene alternative in Section 4.4. Gene counts refer to this comparison-specific sensitivity analysis, not the complete 16-state Group A space.

Table S10. Proteomic detection calls behind the Group A selection. Detection requires a quantified value in at least half of the samples of the dataset concerned: at least 12 of 23 cortical and 10 of 19 medullary feline samples, taken from the cortical and medullary proteome sheets of the published supplementary data of [33]

(42003_2025_9164_MOESM3_ESM.xlsx; the underlying raw data are in ProteomeXchange under PXD066590), where an unquantified sample is blank, and at least 5 of 9 Pod-TRECK samples (Supplementary Data S1), where an unquantified sample is recorded as 0 and a zero is therefore read as not quantified. Symbols were mapped to the human symbol space before intersection. The table below gives the cascade; the per-gene calls for all 22,344 genes of the Δ matrix, with the three detection flags, the Group A or B assignment and whether the gene has finite values in all sixteen states, are supplied as a sheet of the supplementary workbook. Counts are of human symbols after mapping, so they are not protein counts.

Table S11. Observed cosine similarities and descriptive reliability benchmarks for all 120 state pairs. S–B denotes Spearman–Brown. The ratio is observed cosine divided by the S–B benchmark; it is not a bounded cosine estimate and is not used for inference. Shared refers to shared control samples, not all instances of shared animals across tissues. Benchmark estimates and pairwise comparisons are dependent.

Table S12. State-level split-half estimates from 500 partitions. The half-size column retains the recorded case/control counts; for odd groups, it does not by itself specify both partition sizes. S–B ranges are descriptive split-based ranges, not calibrated confidence intervals. Non-positive splits is the proportion with a split cosine at or below zero. Reliability estimates depend on the splitting convention and should be read with Sections 4.10 and 4.11.

Supplementary Figures

Each figure is placed above its legend. The images are the same files as the uploaded figure originals.

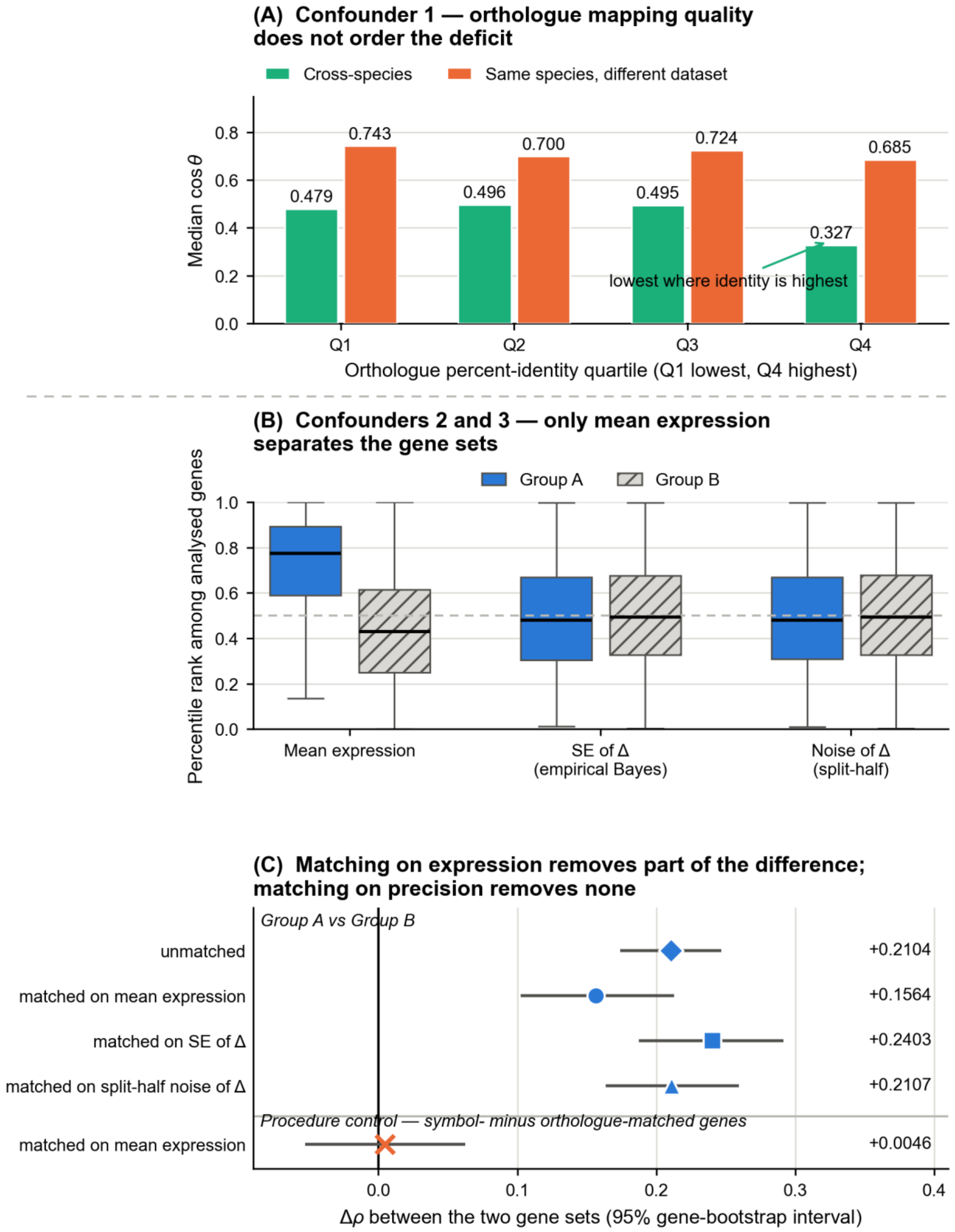

Figure S1. Orthologue characteristics and gene selection. (A) Median cosine in increasing sequence-identity quartiles. Identity measures conservation, not mapping-error rate. (B) Median expression, empirical Bayes error and split-half noise ranks in Groups A and B. Points do not depict the full gene distributions. (C) Group A-minus-Group B differences in cross-species Spearman correlation under the stated matching procedures, with reported central 95% gene-bootstrap ranges. The last row is a separate orthologue-backed versus symbol-matched comparison. These ranges describe gene-resampling sensitivity, not animal-level confidence, and matching does not identify causal contributions.

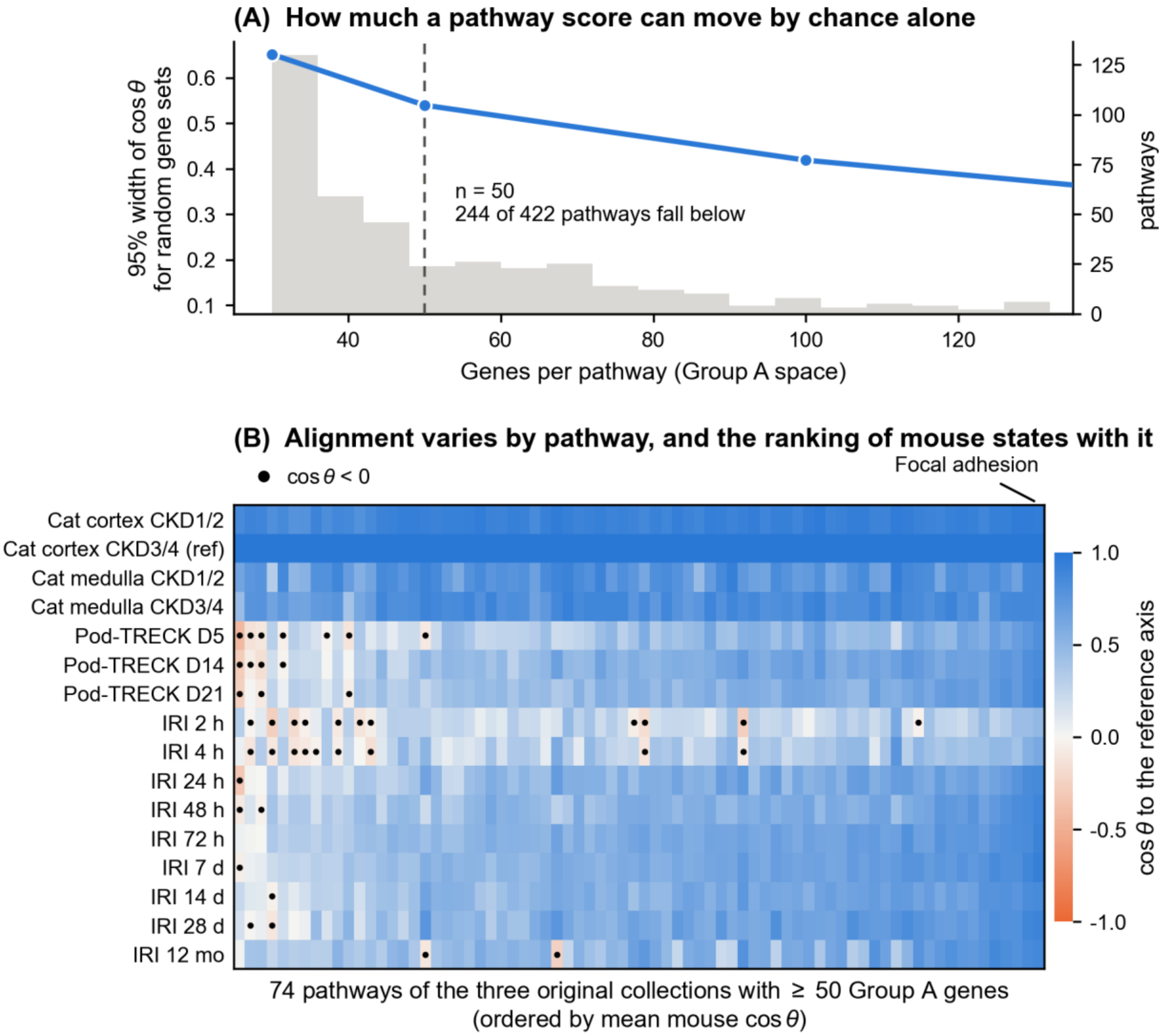

Figure S2. Pathway-level alignment to feline cortical CKD3/4. (A) Central 95% range of cosine values from size-matched random gene sets, with the distribution of pathway sizes. This is a gene-set background distribution, not a sampling interval for a biological pathway. (B) Cosine alignment of 16 states within 74 GO Biological Process, KEGG and Hallmark sets containing at least 50 Group A genes, ordered by mean mouse alignment. Reactome sets are included in the numerical summaries but omitted from this display. Dots mark negative values. Feline profiles share a cohort, and some share control samples, with the reference; their displayed alignment cannot serve as an independent species benchmark.

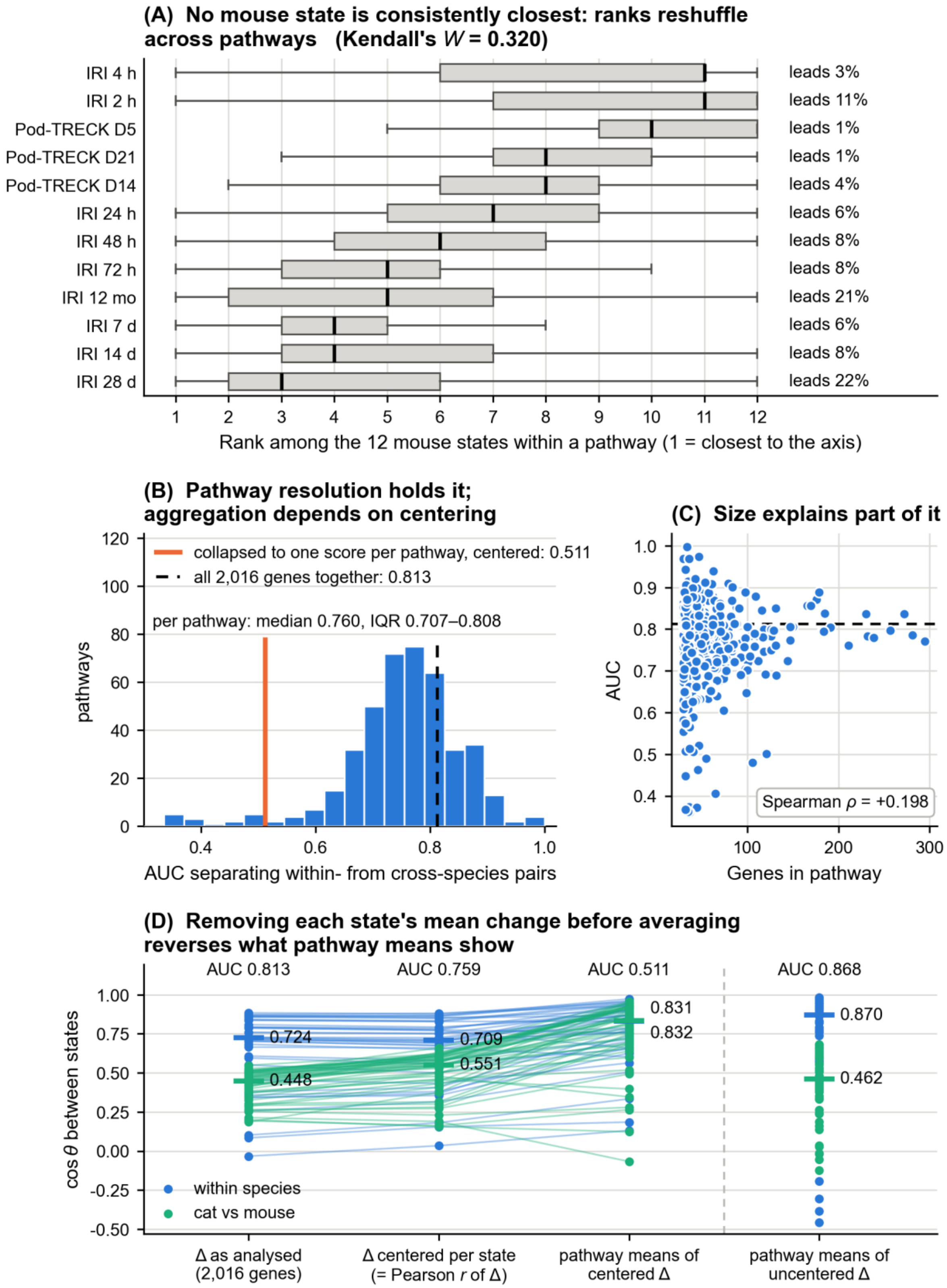

Figure S3. Pathway-specific rankings and the effect of mean-centering on pathway averages. (A) Mouse-state ranks across 422 overlapping pathways; percentages give the share of pathways led by each state. (B) Distribution of pathway-specific AUCs among the 87 state pairs without shared control samples. The dashed line gives the whole-gene AUC and the solid line the AUC after averaging centered responses within pathways. (C) Pathway-specific AUC against gene count. (D) Pairwise cosine under four preprocessing conditions, with class medians and AUCs. Centering is performed within each state across genes, before pathway averaging. Dividing a state by

its own standard deviation does not affect cosine. The plotted AUCs describe these dependent pair classes and are not independent test-set classification accuracies.

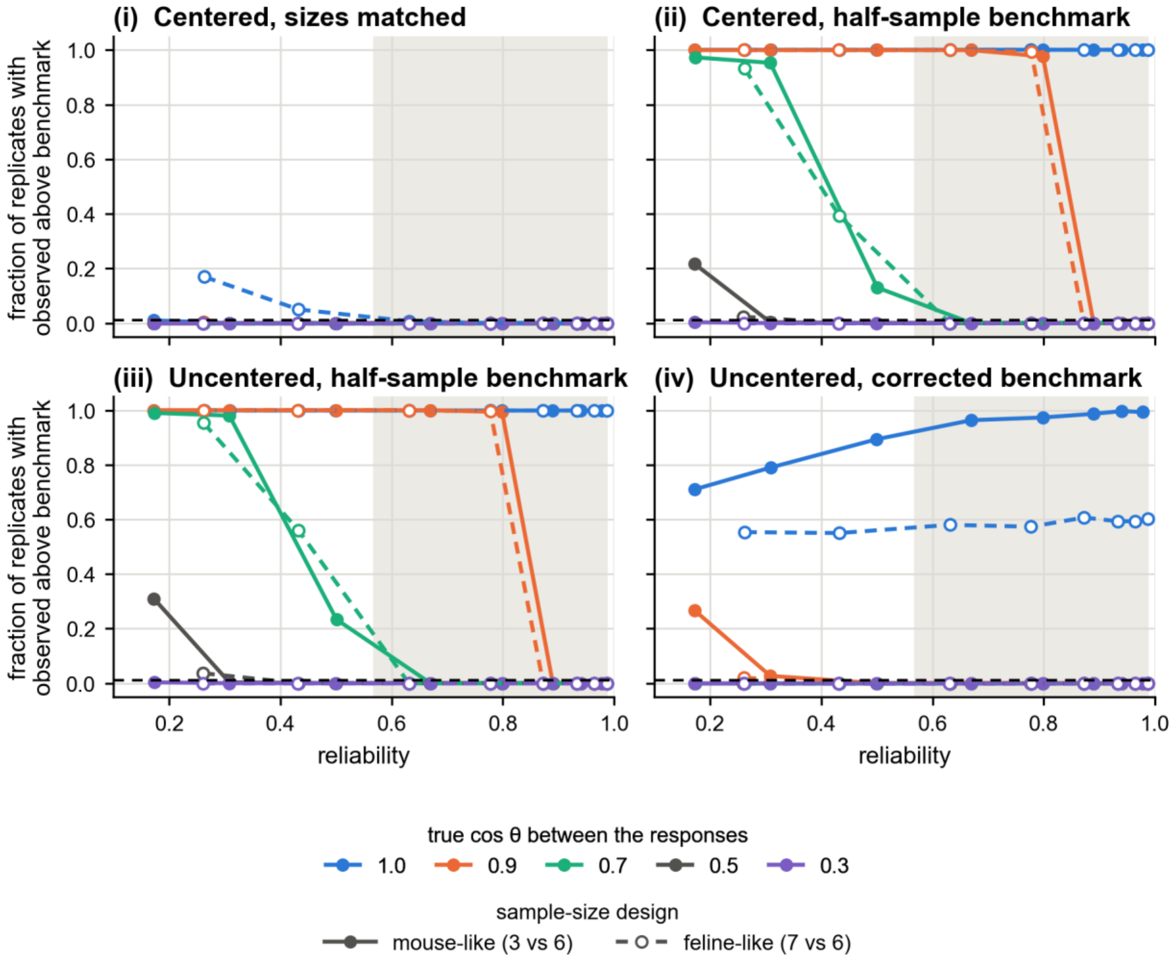

Figure S4. Simulation of observed similarity relative to estimated reliability benchmarks. Panels compare (i) centered correlations with matched sample sizes, (ii) centered correlations using a half-sample benchmark, (iii) uncentered cosines using a half-sample benchmark and (iv) uncentered cosines using the corrected benchmark. Color denotes the underlying cosine between the responses, not an angle in radians. Solid lines with filled markers are the mouse-like design of three cases versus six controls; dashed lines with open markers are the feline-like design of seven versus six. All panels use a common mean shift of 0.26 within-state standard deviations, the simulated value closest to the median empirical shift of 0.257; the rates quoted in Section 4.10 average over all three simulated shifts. The shaded region marks the empirical reliability range. Values outside that region illustrate other simulated conditions and need not have the same exceedance behavior. Benchmark exceedance in this simulation is not a test of a biological difference; calibration of the separate benchmark-gap resampling procedure is reported in Supplementary Note S1.

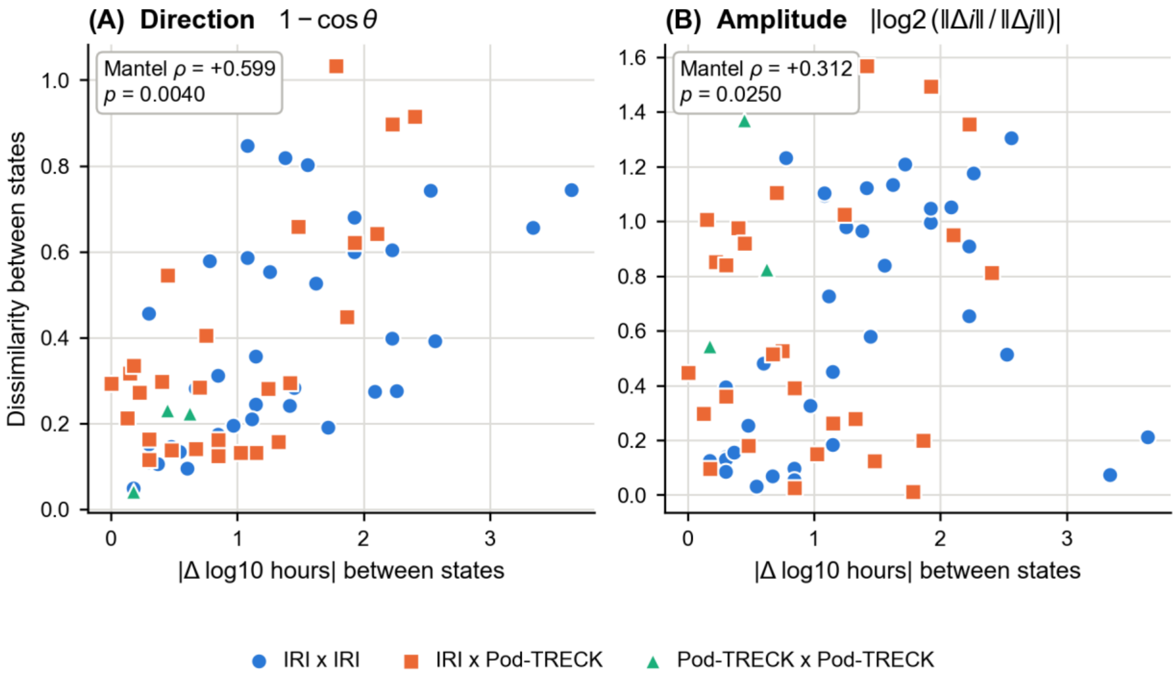

Figure S5. Pairwise directional and amplitude dissimilarity against elapsed time. Each point represents one of the 66 pairs among twelve mouse states. (A) Cosine dissimilarity. (B) Absolute log2 ratio of response norms. Marker shape identifies the model pairing. The inset statistics are exploratory Mantel correlations obtained by permuting state labels, not by treating pairs as independent observations. The stronger directional association in the full panel was less pronounced within IRI alone.
